# Yixin Yangshen Granules Target HIF−1 Signaling to Modulate the Neuroimmune Microenvironment in Alzheimer’s Disease: Insights from Integrative Multi-Omics and Deep Learning

**DOI:** 10.3390/ph19030502

**Published:** 2026-03-18

**Authors:** Zhihao Wang, Linshuang Wang, Yusheng Zhang, Sixia Yang, Bo Shi, Dasheng Liu, Han Zhang, Wan Xiao, Junying Zhang, Xuejie Han, Dongfeng Wei

**Affiliations:** 1 Institute of Basic Research in Clinical Medicine, China Academy of Chinese Medical Sciences, Beijing 100700, China; y13572573172@163.com (Z.W.); wanglinshuang@aliyun.com (L.W.); shibo1521@163.com (B.S.); zzdasheng@163.com (D.L.); 2 Institute of Traditional Chinese Medicine, Tianjin University of Traditional Chinese Medicine, Tianjin 300193, China; 3 Beijing Key Laboratory of Traditional Chinese Medicine Basic Research on Prevention and Treatment for Major Diseases, Experimental Research Center, China Academy of Chinese Medical Sciences, Beijing 100700, China; yushengzhang271727@foxmail.com; 4 School of Traditional Chinese Medicine, Southern Medical University, Guangzhou 510515, China; yangsixia9458@163.com; 5 Guang’anmen Hospital, China Academy of Chinese Medical Sciences, Beijing 100053, China; 6 Clinical Medical College, Jiangxi University of Chinese Medicine, Nanchang 330004, China

**Keywords:** neuroimmune microenvironment, deep learning, Alzheimer’s disease, integrative multi-omics, HIF-1 pathway, AGE-RAGE pathways

## Abstract

**Background/Objectives**: Alzheimer’s disease (AD) involves amyloid and tau pathology with neuroimmune dysregulation, and Yixin Yangshen Granules (YXYS) shows neuroprotective promise, though mechanisms remain unclear. This study aimed to elucidate the multi-target mechanisms of YXYS in AD. **Methods**: The study began by analyzing a public human AD hippocampal snRNA-seq dataset to identify cell-type-specific pathological pathways and profiled YXYS constituents by UPLC-QTOF-MS. In vitro, YXYS cytoprotection against mitochondrial dysfunction and oxidative stress was tested in Aβ_25–35_-challenged HT22 cells; in vivo efficacy was assessed in Aβ_1–42_-induced mice via behavioral and histopathological analyses. Integrated transcriptomic and proteomic profiling of brain tissue, with ELISA, qRT-PCR, and Western blot validation, confirmed pathway targets. Using the intersection of transcriptomic and proteomic targets as biological input, the DTIAM deep learning framework was employed to prioritize active YXYS constituents. Finally, molecular docking and 100-ns dynamics simulations demonstrated direct binding of Ganosporelactone A to HIF−1α. **Results**: AD snRNA-seq analysis highlighted HIF−1 and AGE-RAGE signaling as prominent pathways in the AD hippocampus, particularly enriched in brain microvascular endothelial cells, implicating neurovascular hypoxic and inflammatory stress. In Aβ-induced mice, YXYS improved cognition, reduced Aβ pathology, suppressed neuroinflammation, and promoted neuronal survival, consistent with in vitro evidence of restored mitochondrial function. Multi-omics confirmed convergence on HIF−1 and AGE-RAGE pathways, with YXYS rebalancing the neuroimmune microenvironment by reducing pro-inflammatory M0 macrophages. Screening against these consensus signaling hubs, deep learning analysis prioritized Ganosporelactone A as the top-ranked modulator, and molecular further demonstrated the stable binding of Ganosporelactone A to HIF−1α, linking YXYS to mitigation of hypoxic stress. **Conclusions**: Guided by multi-omics and deep learning, our findings suggest that YXYS may alleviate AD-related phenotypes through multi-target modulation of the HIF−1 and AGE-RAGE pathways, with associated improvements in neuro-immune homeostasis and reductions in oxidative stress, neuroinflammation, and hypoxia.

## 1. Introduction

Alzheimer’s disease (AD) is a complex and progressive neurodegenerative disorder characterized by memory impairment, cognitive decline, and behavioral alterations. Globally, AD affects approximately 30 million people, with estimates suggesting that this number could reach 100 million by 2050 [1].

The neuropathological hallmarks of AD are the extracellular deposition of amyloid-beta (Aβ) plaques and the formation of intracellular neurofibrillary tangles composed of hyperphosphorylated tau protein, which together drive synaptic dysfunction and neuronal loss [2]. Emerging evidence suggests that oxidative stress not only impairs mitochondrial function but also disrupts neuro-immunomodulatory signaling and alters the vascular microenvironment, collectively contributing to the multifactorial pathogenesis of AD [3]. This multifactorial pathogenesis helps explain why current therapeutic agents, which are often directed at single molecular targets, have demonstrated limited efficacy in altering the long-term course of AD, underscoring the urgent need for multi-target therapeutic strategies [4,5].

Recent pharmacological studies have shown that multicomponent herbal formulations can exert significant neuroprotective effects by modulating key molecular targets involved in AD pathology [6,7]. Preclinical studies suggest that these agents may reduce Aβ deposition and tau pathology, mitigate neuroinflammation, and support synaptic remodeling, potentially via mechanisms involving oxidative stress regulation, immune signaling modulation, and cerebrovascular stabilization [8]. These findings highlight the potential of polypharmacology, wherein a combination of bioactive compounds can produce synergistic effects, enhancing the therapeutic outcomes for neurodegenerative conditions such as AD [9,10].

Yixin Yangshen Granules (YXYS), a modern formulation based on traditional Chinese medicine (TCM), comprises a synergistic blend of herbal components. The formula consists of *Panax quinquefolius* L., *Panax notoginseng* (Burkill) F. H. Chen, *Ganoderma lucidum* (Leyss. ex Fr.) Karst, *Cornus officinalis* Sieb. et Zucc, *Artemisia anomala* S. Moore, and *Ligusticum chuanxiong* Hort. Each ingredient offers unique anti-inflammatory, antioxidative, and neuroprotective benefits for AD [11,12,13]. Research on these herbs individually has shown that Panax species can reduce oxidative stress and enhance mitochondrial function, while Ganoderma lucidum exhibits neuroprotective and immunomodulatory effects [14,15]. Grounded in TCM theory, the formulation is designed to tonify ‘qi’ to enhance vitality, nourish ‘Kidney essence’ to support brain health [16], and invigorate blood circulation- concepts that resonate with modern understandings of energy metabolism, neuronal integrity, and cerebrovascular function [17,18,19]. These traditional uses are extensively documented in classical texts and the modern Chinese Pharmacopoeia, providing a strong ethnopharmacological basis for investigating YXYS in the context of AD [20,21,22,23].

Clinical observations further indicate that Yixin Yangshen Granules exert broad and consistent therapeutic benefits in patients with memory decline and cognitive impairment. In routine practice, individuals receiving YXYS frequently demonstrate improvements in attention, memory retrieval, and daily functional performance, reflecting the formula’s capacity to modulate both neurological and systemic pathological mechanisms underlying cognitive disorders. These clinical outcomes align with the formula’s multi-component, multi-target pharmacological profile, supporting its value as an effective complementary therapy for cognitive dysfunction.

Despite preliminary evidence suggesting the potential of YXYS, its specific molecular mechanisms, particularly its integrated effects on the neuro-immune-vascular axis, remain largely uncharacterized. To address this knowledge gap, the present study employed an integrative, multi-scale strategy to systematically delineate the therapeutic actions of YXYS. Our investigation commenced with an analysis of single-cell transcriptomic data from public databases of human AD clinical samples to identify cell-type-specific signatures and core pathological pathways at the highest resolution. Guided by these clinically relevant insights, we subsequently validated the therapeutic efficacy of YXYS in vivo using an Aβ_1–42_ intracerebroventricularly injected mouse model, assessing its effects on cognitive performance and key neuropathological features. To further elucidate the molecular underpinnings, brain tissues from this model were subjected to a multi-omics analysis, incorporating transcriptomics and proteomics. These high-dimensional datasets were then integrated using deep learning algorithms to pinpoint crucial regulatory networks and molecular targets. Subsequently, molecular docking and molecular dynamics simulations were performed to interrogate the binding interactions between the principal bioactive compounds of YXYS and their identified targets at an atomic level. This systematic investigation not only provides robust, multi-layered evidence for the neuroprotective mechanisms of YXYS but also establishes an innovative research paradigm for explaining the complex pharmacology of traditional herbal formulations.

## 2. Results

### 2.1. Single-Nucleus RNA Sequencing and Functional Analysis in AD

#### 2.1.1. Single-Nucleus RNA Sequencing Analysis

**Figure 1:**
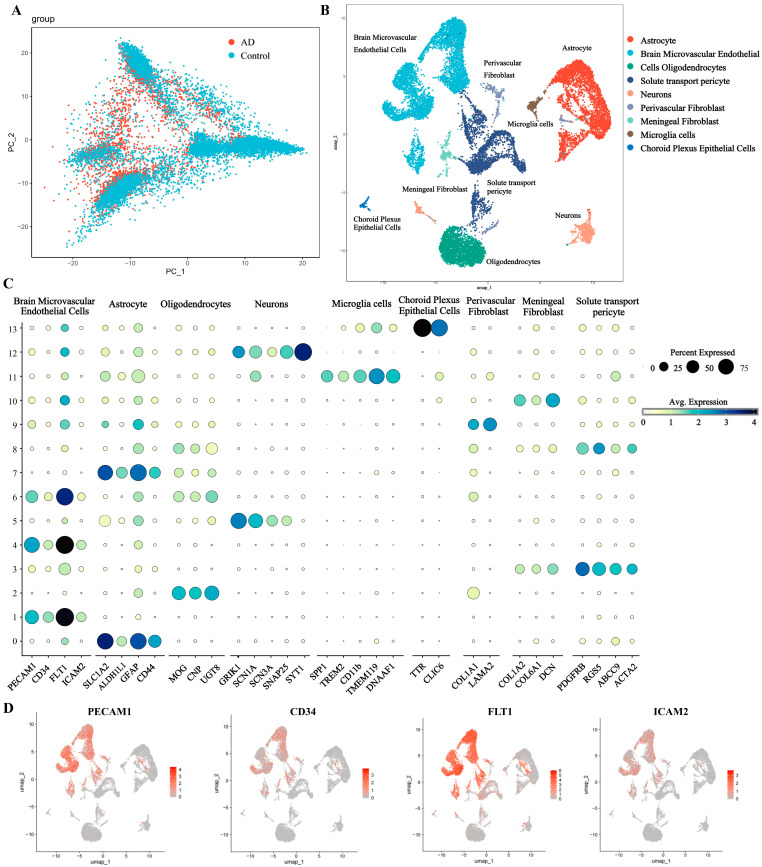
Quality control, dimensionality reduction, and cell type annotation in AD hippocampus from single-nucleus RNA sequencing (snRNA-seq). Data derived from postmortem hippocampal tissue (AD, *n* = 3; NCI controls, *n* = 3). (**A**) Principal Component Analysis (PCA). Scatter plot visualizing the global transcriptomic distribution of the 6 samples on the first two principal components (PC1 and PC2). The lack of distinct separation between AD and NCI groups along the top PCs indicates successful data integration and minimal technical batch effects. (**B**) UMAP Visualization. Uniform manifold approximation and projection (UMAP) plot of 18,656 nuclei, identifying 9 distinct cell populations colored by cell type: astrocytes (blue), BMVECs (red), oligodendrocytes (green), solute transport pericytes (orange), neurons (purple), perivascular fibroblasts (cyan), meningeal fibroblasts (magenta), ependymal cells (yellow), and choroid plexus epithelial cells (black). (**C**) Marker Gene Expression. Dot plot of canonical marker gene expression across clusters; dot size represents the percentage of nuclei expressing the gene, and color scale indicates average expression level. (**D**) Feature Plots. Expression distribution of key endothelial markers (PECAM1, CD34, FLT1, ICAM2) projected onto the UMAP space. (Note: Quality control violin plots have been moved to Supplementary Figure S1 to accommodate the PCA).

After quality control and integration, 18,656 high-quality hippocampal nuclei from AD and NCI hippocampus were retained. PCA of sample-level transcriptomes demonstrated no distinct separation between AD (red) and NCI (blue) groups along PC1 and PC2 (Figure 1A). Clustering identified 9 distinct populations, visualized by UMAP (Figure 1B). A dot plot of marker gene expression confirmed cell type identities across clusters, with average expression scaled by percent expressed (Figure 1C). Feature plots highlighted marker distribution within endothelial cell subclusters (Figure 1D).

**Figure 2:**
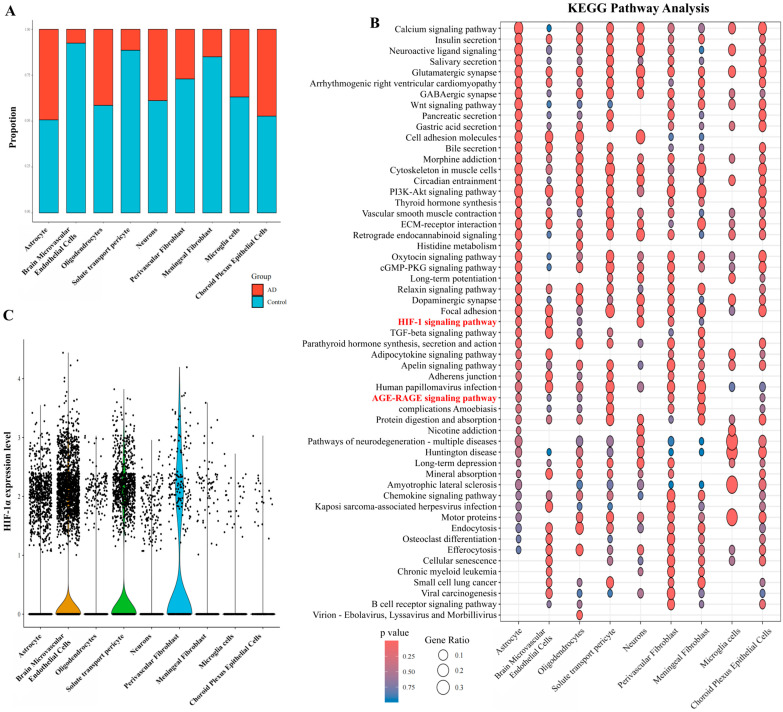
Cellular composition and pathway enrichment in AD hippocampus vasculature. (**A**) Stacked bar plot of cell type proportions across samples, with AD (red) and NCI (blue) segments. (**B**) Bubble plot of top KEGG pathways for DEGs across cell types; bubble size denotes DEG count, color scale indicates −log10 (*p*-value). (**C**) Violin plots of normalized HIF-1α expression across cell types, highest in BMVECs.

A stacked bar plot illustrated cell type proportions across samples (Figure 2A). Group-averaged abundances revealed reduced proportions of brain microvascular endothelial cells (BMVECs) and neurons in AD versus NCI (Wilcoxon rank-sum test). To further visualize sample-level variation, a complementary stacked bar plot depicted the relative proportions of each cell type within representative individual samples (Figure S1A). To further dissect the transcriptional alterations across the identified cell populations, we performed differential gene expression analysis for each of the nine cell types. Differentially expressed genes were visualized in a volcano plot (Figure S1B), with HIF-1α highlighted.

#### 2.1.2. Functional Enrichment Analysis

KEGG analysis showed significant enrichment of the HIF-1 signaling pathway and AGE-RAGE signaling, particularly in BMVECs (Figure 2B). These pathways are critically involved in oxidative stress, inflammatory responses, and cellular adaptation to hypoxia—all hallmarks of AD. This suggests YXYS may act by modulating these stress-response pathways at the cellular level.

GO enrichment of DEGs identified terms related to vascular transport, cell adhesion, and blood–brain barrier integrity (Biological Process); membrane structures like focal adhesions and junctions (Cellular Component); and growth factor/cytokine binding (Molecular Function) (Figure S1C). These suggest disruptions in cellular adhesion and microenvironmental integrity in AD.

#### 2.1.3. Cell Type Specific Expression Patterns of HIF−1α and VEGF-A

Violin plots displayed HIF-1α (Figure 2C) and VEGFA (Figure S1D) expression across populations. HIF-1α was predominantly expressed in BMVECs, aligning with HIF-1 pathway enrichment and indicating endothelial hypoxia signaling in AD.

### 2.2. Chemical Components and Quality Control of the YXYS Formula

**Figure 3:**
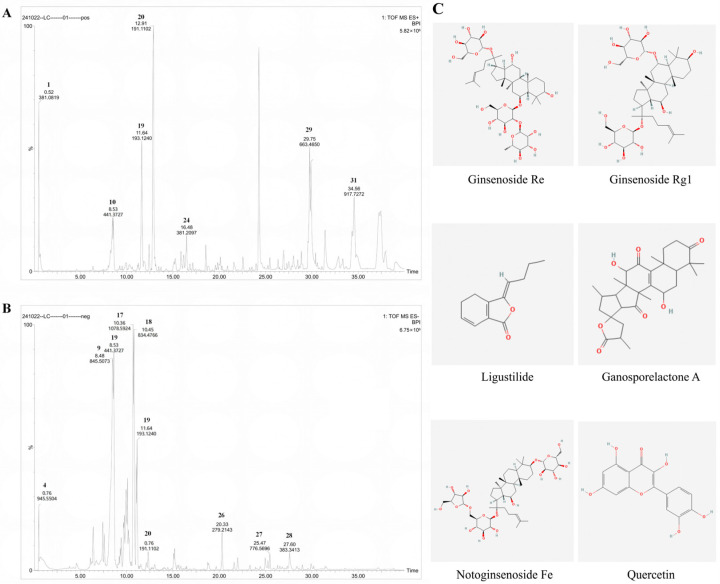
Chemical profiling of YXYS using UPLC-Q-TOF-MS. (**A**) Base Peak Intensity (BPI) chromatogram of YXYS in the positive ion mode. (**B**) BPI chromatogram of YXYS in the negative ion mode. (**C**) Chemical structures of representative bioactive compounds identified in YXYS, illustrating the major chemical classes present in the formula. These include saponins (Ginsenoside Re, Ginsenoside Rg1, and Notoginsenoside Fe), a terpenoid (Ganosporelactone A), a phthalide (Ligustilide), and a flavonoid (Quercetin).

UPLC-QTOF-MS analysis of the YXYS extract tentatively identified 31 compounds (Table 1), including their molecular formulae, retention times (RT), neutral masses, observed m/z values, mass errors, adduct types, response intensities, and CID information. Based on structural features and reported phytochemical classifications, these constituents were grouped into the following chemical classes: Saponins (12 compounds)—including ginsenosides (Rh4, Re, Rg1, Rc, Rs1), notoginsenoside Fe, and other triterpenoid saponins such as marsdenosides A/B, quinquenoside R1, anemoside B, yemuoside YM14, and yesanchinoside E; Terpenoids (8 compounds)—including ganosporelactone A, epi-kansenone, ganoderol A, platycodigenin, ganoderic acids (H, E, A), and one sesquiterpenoid; Flavonoids (3 compounds)—sanggenon A, quercetin, and artemitin; Glycosides (2 compounds)—picroside I and morroniside; Phthalides (2 compounds)—butylphthalide and ligustilide; as well as one coumarin (umbelliferone), one polyacetylene (panaxydol), one tannin (tellimagrandin II), and one phenolic acid (3,4-dihydroxycinnamic acid), as shown in Figure 3A,B.

The identified compounds exhibited RTs ranging from 0.52 min (3,4-dihydroxycinnamic acid) to 34.56 min (notoginsenoside Fe), indicating effective chromatographic separation across a broad polarity window. Mass errors were all within ±10 ppm (from −8.1 ppm for tellimagrandin II to +5.1 ppm for butylphthalide; several features showed very low deviations of ~0.1 ppm, e.g., ginsenoside Rh4, epi-kansenone, and panaxydol), supporting the reliability of the assignments. Overall, the chemical profile of YXYS was characterized by abundant saponins and Ganoderma-derived terpenoids, together with iridoid glycosides, flavonoids, and phthalides, which collectively provided a representative basis for the identity confirmation and quality evaluation of the formula.

Representative chemical structures of bioactive compounds identified in YXYS illustrate the major chemical classes present in the formula, including saponins (Ginsenoside Re, Ginsenoside Rg1, and Notoginsenoside Fe), a terpenoid (Ganosporelactone A), a phthalide (Ligustilide), and a flavonoid (Quercetin), as shown in Figure 3C.

### 2.3. Potential Treatment Mechanism of AD by YXYS Revealed by Network Pharmacology

#### 2.3.1. Active Compounds and Targets Screened and Herb–Compound–Target Network Construction

Based on UPLC-QTOF-MS analysis, 31 bioactive constituents were experimentally identified in the YXYS extract. These empirically verified compounds were then subjected to target prediction, and all candidate targets were standardized against the UniProt database. In total, 619 drug targets were identified. Additionally, 10,849 AD-related targets were screened from GeneCards, OMIM, and DisGeNET databases with a relevance score ≥ 0.3. A total of 496 overlapping targets between YXYS and AD were identified, as shown in the Venn diagrams (Figure S2A,B).

**Figure 4:**
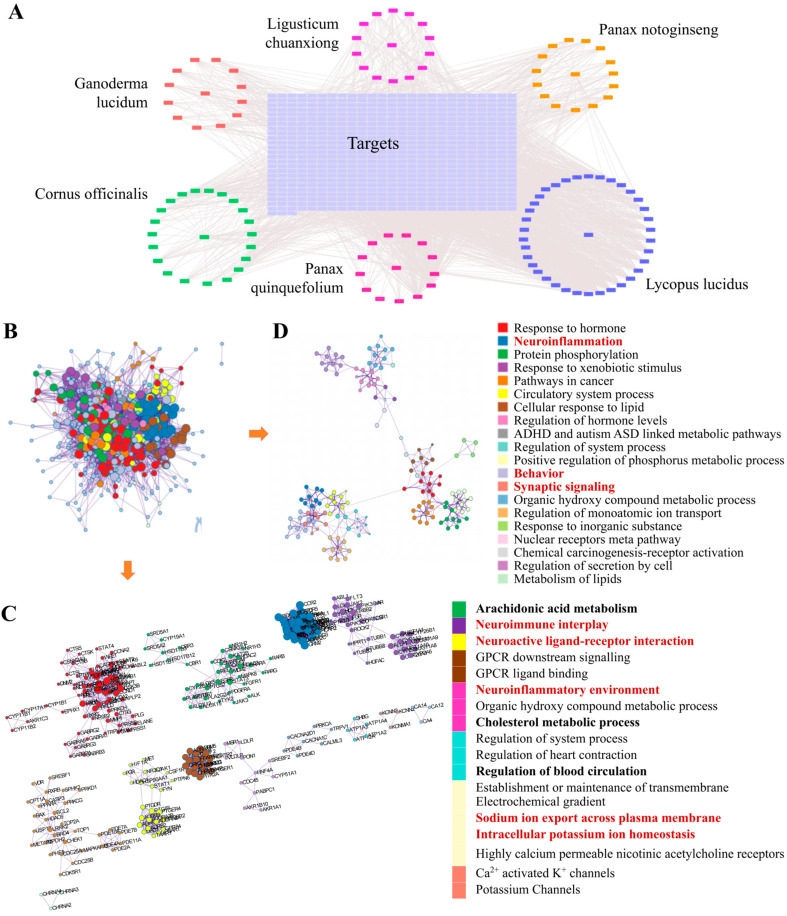
Herb–compound–target network construction and pathway enrichment analysis. (**A**) Herb–compound–target network displaying the relationships between herbal compounds and their associated targets. The square nodes arranged in circles represent the potential compounds corresponding to each herb, while the central square grid nodes represent the screened potential targets. (**B**) Network analysis of enriched pathways based on the shared targets identified between YXYS and AD. Each node represents a specific biological process, and edges indicate the relationships between these processes. (**C**) Network diagram of key biological processes associated with AD pathogenesis, annotated with cluster categories. (**D**) Network diagram of significantly enriched pathways, annotated with cluster categories.

To visualize the relationships among compounds, targets, and herbs, a herb–compound–target network was constructed (Figure 4A). The central circular area represents overlapping compounds among the six herbs, with overlapping targets highlighted in the central purple square region.

#### 2.3.2. Functional Analysis of Common Targets

These 496 overlapping targets may be potential therapeutic targets of YXYS in AD. A protein–protein interaction (PPI) network was constructed to reveal the interactions among these proteins (Figure 4B), and functional clusters containing proteins with similar functions were identified and represented by different colors (Figure 4C). These clusters were related to neuroinflammation, neuroactive ligand–receptor interactions, the neuroinflammatory environment, cholesterol metabolism, and ion homeostasis and transport. The KEGG enrichment analysis results were clustered according to function (Figure 4D). The main enriched pathways included neuroinflammation, synaptic signaling, cellular responses to lipids, and lipid metabolism. The GO terms enriched in biological processes, cellular component categories, and molecular function categories are shown in Figure S1C. The main biological processes included biological regulation, metabolic processes, cell communication, and cellular component organization. Cellular components included protein-containing complexes, vesicles, endoplasmic reticulum, and mitochondria. Its main molecular functions included oxygen binding, antioxidant activity, ion binding, transporter activity, enzyme regulation, and electron transfer activity. YXYS may enhance vascular endothelial function by modulating cholesterol and lipid metabolism, thereby improving cerebral circulation. These results indicate that YXYS could regulate cellular communication and metabolism, maintaining redox balance and antioxidant activity, which may mitigate AD-related cellular damage and neuroinflammation. In summary, YXYS exerts its therapeutic effects by modulating neuroinflammation, cellular stress responses, and metabolic processes, collectively influencing the progression of AD.

### 2.4. Cell Viability Assessment and Serum Concentration Selection

**Figure 5:**
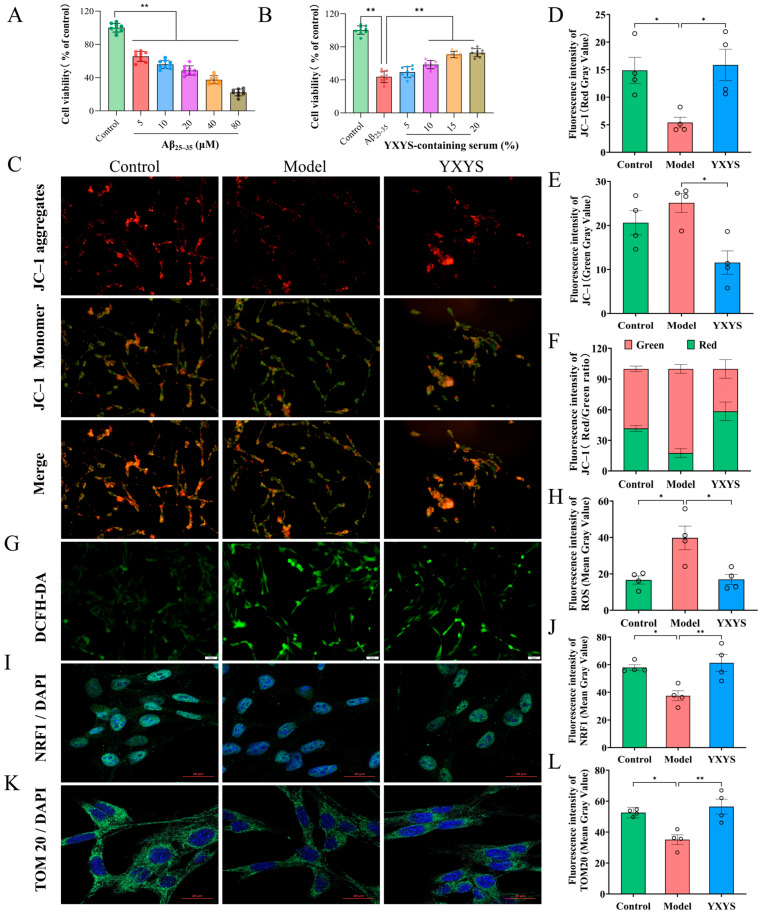
Analysis of Cell Viability, Mitochondrial Membrane Potential, and ROS Fluorescence. (**A**) Effects of various Aβ_25–35_ concentrations on cell viability. (**B**) Effects of different concentrations of YXYS-containing serum on cell viability. (**C**) JC-1 fluorescence images for the control, model, and YXYS groups. Scale bar = 50 μm. (**D**) Quantitative analysis of JC-1 red fluorescence intensity. Red fluorescence corresponds to JC-1 aggregates, with higher intensity indicating higher mitochondrial membrane potential. (**E**) Quantitative analysis of JC-1 green fluorescence intensity. Green fluorescence corresponds to JC-1 monomers, where increased intensity indicates reduced mitochondrial membrane potential and mitochondrial dysfunction. (**F**) Statistical analysis of the red/green fluorescence intensity ratio from JC-1 staining across groups. (**G**) Representative images of DCFH-DA staining for intracellular ROS detection. Scale bar = 50 μm. (**H**) Quantitative analysis of average ROS fluorescence intensity. (**I**) NRF1 expression results, scale bar = 20 μm. (**J**) Quantitative analysis of average NRF1 fluorescence intensity. (**K**) TOM20 expression results, scale bar = 20 μm. (**L**) Quantitative analysis of average TOM20 fluorescence intensity. (* *p* < 0.05; ** *p* < 0.01. Data are presented as mean ± SD, *n* = 3).

To investigate the neuroprotective properties of YXYS against Aβ_25–35_–induced cellular damage, cell viability was measured using the CCK-8 assay (Figure 5A,B). Cytotoxicity assays revealed that Aβ_25–35_ treatment significantly reduced cell viability compared to the control group (*p* < 0.05); accordingly, 20 µmol/L Aβ_25–35_ was selected as the condition for model establishment. In neuroprotection assays, intervention with YXYS–medicated serum significantly restored the viability of Aβ_25–35_–injured cells compared to the model group (*p* < 0.05). Consequently, 10% YXYS-medicated serum was selected as the YXYS intervention condition for subsequent experiments. These results demonstrate that YXYS significantly alleviates Aβ_25–35_–induced cytotoxicity.”

### 2.5. YXYS Protects HT22 Cells from Aβ_25–35_-Induced Mitochondrial Dysfunction and Oxidative Stress

#### 2.5.1. YXYS Alleviates Aβ_25–35_-Induced Mitochondrial Dysfunction in HT22 CELLS

The status of mitochondrial membrane potential (MMP) was assessed through JC-1 staining (Figure 5C). In untreated cells, JC-1 dye predominantly accumulated in mitochondria as red-fluorescent aggregates, reflecting intact MMP. Conversely, treatment with Aβ_25–35_ led to a shift from red to green fluorescence, indicative of mitochondrial depolarization (*p* < 0.05; Figure 5D,E). Quantitative analysis showed a significant reduction in the red-to-green fluorescence ratio in the model group (*p* < 0.05; Figure 5F). Remarkably, administration of YXYS-medicated serum restored MMP, as demonstrated by enhanced red fluorescence and a notable increase in the red/green fluorescence ratio relative to Aβ-treated cells (*p* < 0.05). These observations suggest that YXYS stabilizes mitochondrial function and prevents depolarization caused by Aβ_25–35_ exposure.

#### 2.5.2. YXYS Reduces Intracellular ROS Levels in HT22 Cells

To explore the antioxidative potential of YXYS, intracellular reactive oxygen species (ROS) were measured using DCFH-DA fluorescence staining (Figure 5G,H). Aβ_25–35_ exposure resulted in a significant elevation of ROS generation compared to the control group (*p* < 0.05). Treatment with YXYS-medicated serum markedly reduced ROS accumulation (*p* < 0.05), indicating its capacity to counteract Aβ_25–35_-induced oxidative stress in neuronal cells.

#### 2.5.3. YXYS Enhances NRF1 Expression in Aβ_25–35_-Treated HT22 Cells

To further elucidate the protective mechanism of YXYS, NRF1 expression was examined via immunofluorescence staining (Figure 5I,J). Quantitative analysis of fluorescence intensity revealed that Aβ_25–35_ significantly reduced NRF1 expression compared to the control group (*p* < 0.05). However, treatment with YXYS-medicated serum effectively restored NRF1 expression (*p* < 0.01), suggesting that YXYS exerts its protective effects by regulating mitochondrial biogenesis.

#### 2.5.4. YXYS Restores Mitochondrial Biogenesis Marker TOM20 in HT22 Cells

Mitochondrial biogenesis was further evaluated by detecting TOM20 expression via immunofluorescence staining (Figure 5K,L). Compared to the control group, Aβ_25–35_ exposure significantly reduced TOM20 expression (*p* < 0.05). However, YXYS-medicated serum treatment significantly increased TOM20 expression levels compared to the model group (*p* < 0.01), indicating that YXYS promotes mitochondrial biogenesis in Aβ_25–35_-treated HT22 cells.

### 2.6. Improved Cognitive Ability in Aβ Mice Following Treatment with YXYS

**Figure 6:**
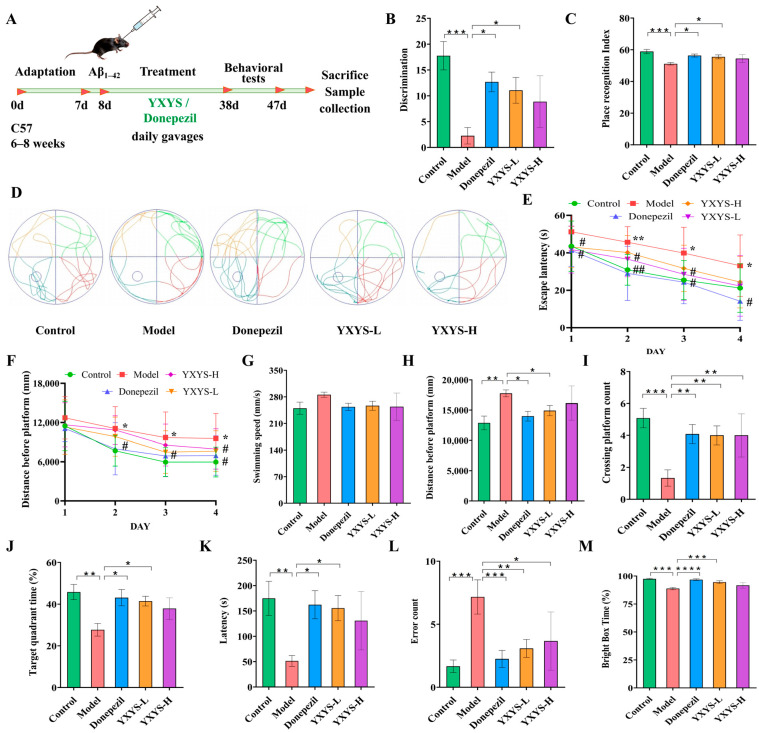
Improved cognitive function in Aβ mice after YXYS treatment. (**A**) Experimental timeline of treatment and behavioral tests. C57 mice were injected with Aβ and treated daily with YXYS or donepezil. Behavioral tests, including the NOR test, passive avoidance test, and MWM, were performed between days 60 and 67. (**B**) Discrimination index (DI) in the NOR test. (**C**) Place recognition index (PRI) in the NORT. (**D**) Representative movement trajectories of mice during the MWM probe test. (**E**) Escape latency during the hidden platform training phase of the MWM test over four days. (**F**) Distance traveled before reaching the platform during the MWM test. (**G**) Swimming speed. (**H**) Distance traveled before platform crossing during the probe trial. (**I**) Number of platform crossings during the MWM probe test. (**J**) Percentage of time spent in the target quadrant during the MWM probe test. (**K**) Latency to enter the dark compartment in the passive avoidance test. (**L**) Error count (number of shocks) in the passive avoidance test. (**M**) Time spent in the bright compartment during the passive avoidance test. All data are presented as mean ± SD. (*n* = 12) * *p* < 0.05, ** *p* < 0.01, *** *p* < 0.001, **** *p* < 0.0001, ^#^ *p* < 0.05, ^##^ *p* < 0.01.

The in vivo efficacy of YXYS was assessed through a series of behavioral assays, with the experimental layout and timeline depicted in Figure 6A. Recognition memory was assessed using the novel object recognition (NOR) test. As presented in Figure 6B, Aβ-injected mice exhibited marked reductions in both the discrimination index (DI) and novel object preference compared to control animals (*p* < 0.05), indicative of cognitive impairment. Notably, mice treated with YXYS-L (4.68 g/kg) and YXYS-H (9.36 g/kg) or donepezil showed significant improvements in these metrics relative to the model group (*p* < 0.05). YXYS administration notably increased the duration of novel object exploration (Figure 6C), suggesting restored recognition memory and enhanced retention. Figure 6D outlines the workflow of the Morris water maze (MWM) test, which was employed to assess spatial learning and memory. During the training phase (days 2–4), all groups demonstrated progressive reductions in escape latency, reflecting learning acquisition (Figure 6E,F). However, mice in the Aβ group required significantly more time to locate the hidden platform compared to controls (*p* < 0.01), indicating impaired spatial learning. Treatment with YXYS significantly shortened escape latency relative to the model group (*p* < 0.05), suggesting improved learning capacity.

In the probe trial performed on day 5, swimming speeds were comparable across all groups (Figure 6G), ruling out motor deficits. Mice treated with YXYS-L showed increased frequency of platform zone crossings (Figure 6H,I) and spent more time in the target quadrant than untreated Aβ mice (*p* < 0.05, Figure 6J), indicating that spatial memory was significantly preserved by YXYS.

The passive avoidance test was conducted to examine aversive memory retention. Aβ-challenged mice exhibited a significant reduction in latency to enter the dark compartment and received a higher number of electric shocks, consistent with memory deficits (*p* < 0.01). In contrast, mice administered YXYS-L showed prolonged latencies before entering the dark compartment (Figure 6K) and made fewer errors (Figure 6L), with both being significantly improvements compared to the model group (*p* < 0.05). Moreover, the time spent in the light compartment was significantly increased YXYS-L and YXYS-H group (Figure 6M), indicating enhanced memory retention and a potential anxiolytic effect. Collectively, these results suggest that YXYS improves both cognitive and emotional outcomes in Aβ-induced neurodegeneration.

### 2.7. HE and Nissl Staining in Aβ Mice Following Treatment with YXYS

**Figure 7:**
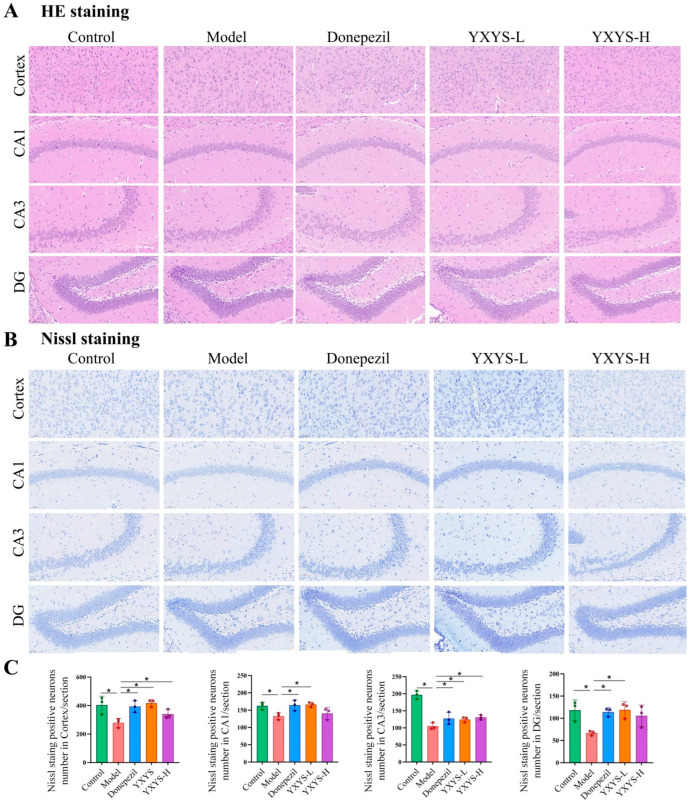
Ameliorated pathological changes in the brains of Aβ mice after YXYS treatment. (**A**) HE staining of the cortex, Cornu Ammonis (CA1), CA3, and dentate gyrus (DG) in control, model, donepezil, and YXYS-L and YXYS-H groups. The model group exhibits cellular disorganization and neuronal loss, which is ameliorated by YXYS and donepezil treatments (magnification, 30×). (**B**) Nissl staining of the same regions, showing reduced Nissl-positive neurons in the model group, with improved neuron density in the YXYS and donepezil groups (magnification, 30×). (**C**) Quantification of Nissl-positive neurons in each region (cortex, CA1, CA3, and DG). Data are presented as mean ± SD (*n* = 3). * *p* < 0.05.

Histopathological evaluation using HE staining revealed pronounced neuronal damage in Aβ-induced mice, with signs of disorganization and neuronal loss in the cortex, CA1, CA3, and DG regions of the hippocampus (Figure 7A). In contrast, mice treated with YXYS or donepezil showed improved histological structures, reduced vacuolation, and more intact cellular layers. Nissl staining confirmed these results, showing fewer Nissl-positive neurons in the model group (*p* < 0.05) (Figure 7B). YXYS-L and YXYS-H treatment significantly increased the number of Nissl-positive neurons across all regions, with the greatest effect observed in the DG, exceeding that of the donepezil (*p* < 0.01). These results suggest that YXYS provides neuroprotection against Aβ-induced damage, potentially outperforming donepezil in some areas. After systematically evaluating the efficacy and safety profiles across different doses, the low-dose group (4.68 g/kg) was chosen for subsequent multi-omics analyses. 

### 2.8. Transcriptomics Analysis

#### 2.8.1. Quantitative Analysis and Differential Gene Analysis

To further explore the molecular mechanisms by which YXYS regulates the pathological changes in Aβ mice, the FPKM values for all sample genes were calculated and visualized using box plots. Correlation coefficients and Principal Component Analysis (PCA) results-PC1 (21.51%) and PC2 (17.45%)-revealed significant inter-sample variability and good intra-sample reproducibility (Figure S3A).

Figure S3B presents a volcano plot of DEGs, illustrating the significant changes in gene expression between the treatment and control groups. In the comparisons among the control, model, and YXYS groups, a total of 979 DEGs were identified. Relative to the control group, 297 genes were upregulated and 144 were downregulated. In comparison with the model group, 180 genes were upregulated and 100 were downregulated (Figure S3C).

#### 2.8.2. GO and KEGG Pathway Analysis of DEGs

Functional enrichment analyses of DEGs explored the molecular mechanisms of YXYS in AD treatment. DEGs were significantly enriched in pathways related to peptide cross-linking, vesicle fusion regulation, sympathetic nervous system development, ROS regulation, and metabolic processes. In particular, processes such as the response to drugs and positive regulation of ROS metabolic processes were prominently represented, suggesting that YXYS may exert therapeutic effects by regulating oxidative stress and neuroinflammation, both of which are critical factors in AD progression.

In the functional category analysis using the Cluster of Orthologous Groups (COG) database (Figure S3D), several relevant categories emerged in the context of AD, particularly energy production and conversion, post-translational modification, protein turnover, and chaperones. Energy metabolism is crucial for neuronal health, and its impairment is linked to neurodegeneration in AD. Furthermore, post-translational modifications are vital for regulating protein function and stability. Proper protein turnover and chaperone activity are essential for preventing the accumulation of misfolded proteins associated with amyloid plaques. These findings suggest that targeting these functions may enhance neuronal resilience and slow AD progression by improving protein and energy homeostasis.

In the biological process category, the DEGs were significantly enriched in pathways associated with drug response, axoneme assembly, and peptide cross-linking. Particularly, processes such as response to drugs and positive regulation of ROS metabolic processes were prominently represented, suggesting that YXYS may exert therapeutic effects by regulating oxidative stress and neuroinflammation, both of which are critical factors in AD progression (Figure S3E).

In the cellular component category, the significantly enriched terms included extracellular space, collagen-containing extracellular matrix, and motile cilia. Enrichment in the extracellular matrix (ECM) and related components highlights the importance of maintaining structural integrity in the brain. These findings suggest that YXYS may help preserve the integrity of the extracellular environment, potentially preventing synaptic loss and neurodegeneration typically seen in AD (Figure S3F).

In the molecular function category, significantly enriched terms included fibrinogen binding and calcium-dependent protein binding. These binding activities are crucial for cellular communication, signaling, and homeostasis within the neural microenvironment. The modulation of these functions suggests that YXYS may promote synaptic stability and improve neuronal signaling through regulation of PPI and calcium homeostasis (Figure S3G).

**Figure 8:**
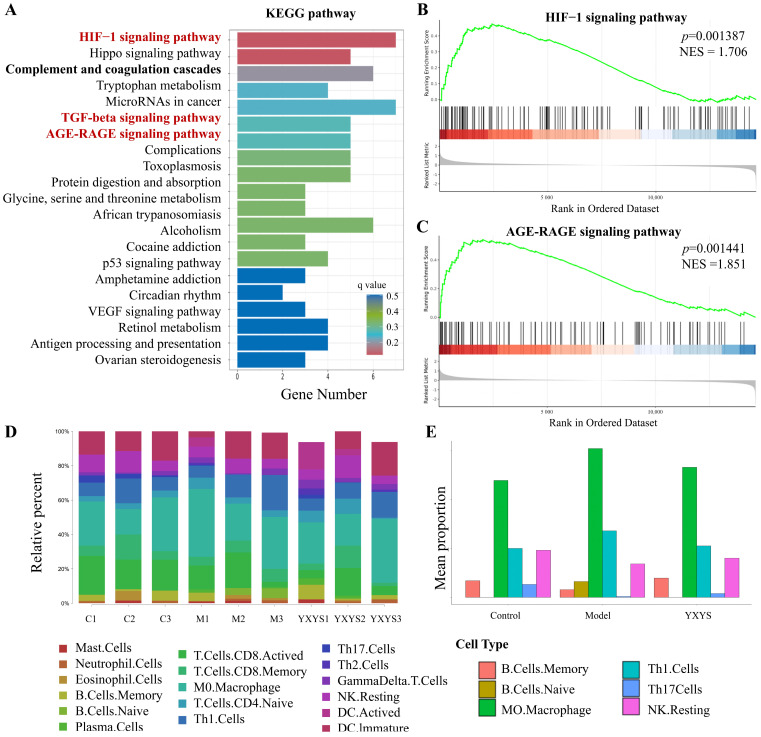
Pathway enrichment and gene set analysis. (**A**) KEGG pathway enrichment analysis of DEGs, revealing significant enrichment in pathways such as HIF-1, TGF-beta, AGE-RAGE, and VEGF signaling pathways, suggesting their roles in AD pathology. (**B**) GSEA plot for the HIF-1 signaling pathway. (**C**) Gene Set Enrichment Analysis (GSEA) plot for the AGE-RAGE signaling pathway. (**D**) Stacked bar plot showing the relative proportions of immune cell types in each group, reflecting changes in immune cell infiltration. (**E**) Histogram highlighting the major immune cell types influenced by YXYS treatment.

KEGG pathway analysis revealed several enriched signaling pathways that are crucial for understanding the therapeutic mechanisms of YXYS (Figure 8A). Among the top enriched pathways were the HIF-1, AGE-RAGE, TGF-beta, p53, and VEGF signaling pathways, all of which are highly relevant to neurodegenerative processes.

#### 2.8.3. GSEA Enrichment Analysis Results

The HIF-1 signaling pathway showed a moderate enrichment score (NES = 1.706, *p* = 0.001) (Figure 8B and Figure S4A). Within this pathway, genes such as Cdkn1a (involved in cell-cycle regulation) and Hmox1 (associated with oxidative stress response) are relevant to AD pathology. Their differential expression in response to YXYS treatment suggests potential neuroprotective effects by enhancing metabolic adaptation and reducing hypoxic stress in the brain. HIF-1 serves as a central regulator of cellular responses to hypoxia, indicating that YXYS may alleviate hypoxic conditions, promoting neuronal survival and decreasing apoptosis.

The AGE-RAGE signaling pathway was significantly enriched (NES = 1.851, *p* = 0.01341) (Figure 8C and Figure S4B), indicating its potential involvement in the therapeutic effects of YXYS. Key genes such as Icam1 (involved in inflammatory responses) and Mmp2 (associated with ECM remodeling) have emerged as key factors in AD pathology. Their modulation in response to YXYS treatment suggests that this pathway may play a role in mitigating neuroinflammation and enhancing tissue repair. Additionally, genes such as Col1a1 and Col3a1, which are critical for collagen synthesis and ECM integrity, indicate that YXYS may help maintain structural support in the brain, potentially counteracting neurodegeneration. The involvement of Akt2, a key regulator of cell survival and metabolism, further suggests that YXYS may enhance neuronal resilience through pathways associated with survival and repair. Overall, the significant enrichment of the AGE-RAGE signaling pathway highlights its relevance in promoting neuroprotection and modulating inflammatory processes in the context of AD. These findings position YXYS as a promising therapeutic agent.

The TGF-beta signaling pathway also demonstrated significant enrichment (NES = 1.505, *p* = 0.01341) in Figure S4C. Key genes, such as Tgfb3 and Smad5, which are involved in cellular signaling and transcriptional regulation, indicate that YXYS may influence critical processes related to cell growth, differentiation, and response to injury. Additionally, genes, such as Thbs1 and Fmod, which regulate ECM remodeling and fibrosis, suggest that YXYS may help maintain tissue integrity and reduce pathological changes in the brain. The presence of Id3 and Id1, which are involved in the regulation of cell-cycle progression and differentiation, further supports the hypothesis that YXYS enhances neuronal survival and mitigates neurodegeneration. The significant enrichment of the TGF-beta signaling pathway highlights its importance in modulating cellular responses and maintaining homeostasis in the brain, reinforcing the potential of YXYS as a therapeutic agent in AD.

The VEGF signaling pathway exhibited a modest enrichment score (NES = 1.095) but did not reach statistical significance (*p* = 0.3282) (Figure S4D). VEGF is essential for angiogenesis and vascular health maintenance, which are important factors in preventing AD-related cerebrovascular deficits. Although the pathway did not reach significance, the observed trend suggests that YXYS may contribute to improved cerebral vascular function and enhanced oxygen and nutrient delivery to brain tissue.

### 2.9. Characteristics of Immune Cell Infiltration

The immune cell composition was estimated using ImmuCC, a validated mouse-specific immune deconvolution framework, based on normalized transcriptomic profiles. Initially, the data were deconvoluted using a known immune cell marker gene matrix. This allowed for a comprehensive assessment of immune cell types among the groups, as illustrated in the bar chart showing the distribution proportions of each immune cell type.

A heatmap illustrates the correlations between various immune cell types across all experimental groups (Figure S4E). Notably, in the AD model group, M0 macrophages showed a strong negative correlation with resting natural killer (NK) cells (r = −0.81) and CD8^+^ T cells (r = −0.78). This suggests a suppressed immune response and increased inflammation within the AD brain. In contrast, the YXYS group demonstrated improved correlations, particularly between memory B cells and macrophages, indicating a potential restoration of immune regulation following treatment. The bar chart (Figure 8D) compares the relative proportions of immune cells across the control (C1-C3), AD model (M1-M3), and YXYS-treated groups (YXYS1-YXYS3). The AD model group displayed a higher proportion of M0 macrophages and neutrophils, indicating a pro-inflammatory environment. Following YXYS treatment, a marked increase in CD8^+^ T, memory B, and resting NK cells was observed, reflecting enhanced immune surveillance and a reduced inflammatory response.

Quantitative immune profiling indicated a marked increase in M0 macrophage abundance within the AD model group compared to both the control and YXYS-treated groups (*p* < 0.05; Figure 8E), reflecting an intensified neuroinflammatory state. Notably, administration of YXYS led to a significant decline in M0 macrophage levels, accompanied by a concurrent rise in Th1 cells, memory B cells, and resting natural killer (NK) cells (*p* < 0.05). These alterations in immune cell populations imply that YXYS contributes to restoring immune equilibrium, fosters the activation of adaptive immune components, and potentially strengthens neuroimmune defense mechanisms.

These findings highlight the potential of YXYS to modulate immune cell infiltration in the AD brain by attenuating the pro-inflammatory environment while enhancing immune homeostasis and neuroprotection. The observed changes in immune cell composition may significantly contribute to the therapeutic effects of YXYS in mitigating AD pathology, emphasizing its role in enhancing immune regulation under neurodegenerative conditions.

### 2.10. Proteomic Analysis Reveals Potential Therapeutic Targets of YXYS in AD Treatment

Using mass spectrometry and proteomic data analysis, a total of 5028 proteins were identified in the brain tissues from the control and model groups. DEPs were identified from the quantifiable datasets after applying a linear transformation for normalization. PCA results clearly demonstrated significant differences among the control (blue), model (red), and YXYS-treated groups (green) (Figure S5A). The distinct separation of the control group from both the model and YXYS groups along PC1, PC2, and PC3 indicates substantial differences in protein expression and other measured characteristics. Notably, the separation between the YXYS and model groups suggested that the therapeutic intervention had a significant impact on the AD model, potentially reversing or modifying the abnormal expression characteristics observed in the model group.

**Figure 9:**
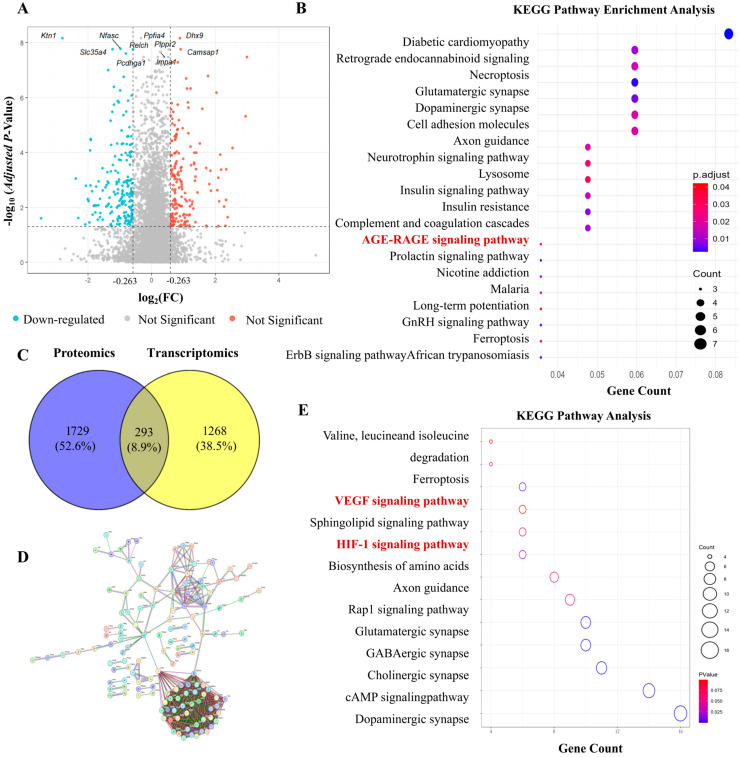
Integrated multi-omics analysis results. (**A**) Volcano plot of DEPs. Red dots indicate upregulated genes, blue dots indicate downregulated genes, and gray dots represent genes with no significant change. (**B**) KEGG pathway enrichment analysis of DEPs. (**C**) Venn diagram showing the overlap between differentially expressed proteins (proteomics) and genes (transcriptomics). A total of 293 overlapping targets were identified between the proteomic and transcriptomic analyses. (**D**) the PPI network built from intersecting target genes. (**E**) KEGG pathway enrichment analysis highlighting key pathways.

Comparative analysis between the YXYS-treated and model groups revealed 2208 DEPs, of which 842 proteins were upregulated and 1366 proteins were downregulated (FDR < 0.05). In the control group versus model group comparison, 1041 DEPs were identified, of which 628 proteins were upregulated and 413 downregulated (FDR < 0.05), as illustrated in Figure S5B. Figure 9A presents a volcano plot illustrating the protein expression differences between the control and model groups. Genes, such as Serpina3k, Plekha5, and Rapgef2, showed pronounced expression differences between the two groups. Genes positioned on the right are upregulated in the model group, whereas those on the left are downregulated in the control group. The horizontal and vertical lines in the plot indicate the significance and FC thresholds, respectively.

Based on the screening results, proteins with similar expression trends between the control vs. model and YXYS vs. model comparisons were filtered out (Figure S5C). The KEGG pathway analysis indicated that these DEPs were associated with the AGE-RAGE signaling pathway and other AD-related pathways (Figure 9B). GO functional enrichment analysis was also conducted (Figure S5D). In terms of biological processes, the DEPs were mainly involved in chemical synaptic transmission, energy reserve metabolic processes, anterograde transsynaptic signaling, and regulation of low-density lipoprotein particle receptors. From the perspective of cellular components, these proteins primarily participate in neuronal projections, dendrites, and lysosomes. Regarding molecular function, most DEPs were localized to amyloid-beta binding, glutamate receptor activity, postsynaptic membrane potential regulation, and iron- and calcium-binding sites.

### 2.11. Integrated Multi-Omics Analysis Results

Differential analysis of transcriptomics and proteomics results was conducted (*p* < 0.05), followed by the construction of a Venn diagram to visualize the intersecting genes, as shown in Figure 9C. This analysis revealed intersecting genes that were subsequently subjected to PPI analysis, resulting in the generation of a protein interaction network (Figure 9D). PPI network analysis identified AKT2, HIF-1α, and VEGF as central regulatory nodes. This result supports the hypothesis that YXYS mediates its effects through coordinated regulation of key pathways involved in cellular resilience and inflammatory responses. KEGG pathway analysis (Figure 9E) identified significant enrichment in the HIF-1 and VEGF signaling pathways, as well as pathways related to ferroptosis and synaptic transmission. These findings suggest that YXYS contributes to the attenuation of neuroinflammation, oxidative stress reduction, and the preservation of synaptic integrity.

### 2.12. Deep Learning-Guided Prioritization Identifies Ganosporelactone A as a Dual Modulator of HIF−1α and RAGE Signaling

While our multi-omics and experimental data have implicated the HIF-1 signaling pathway and the AGE-RAGE axis in the therapeutic mechanism of YXYS, identifying the precise “anchor molecules” responsible for driving these effects from the complex formula remains a challenge. To address this, we employed DTIAM, a cutting-edge deep learning framework pre-trained on massive molecular data, to systematically screen YXYS-derived compounds against the core therapeutic target HIF-1α.

**Figure 10:**
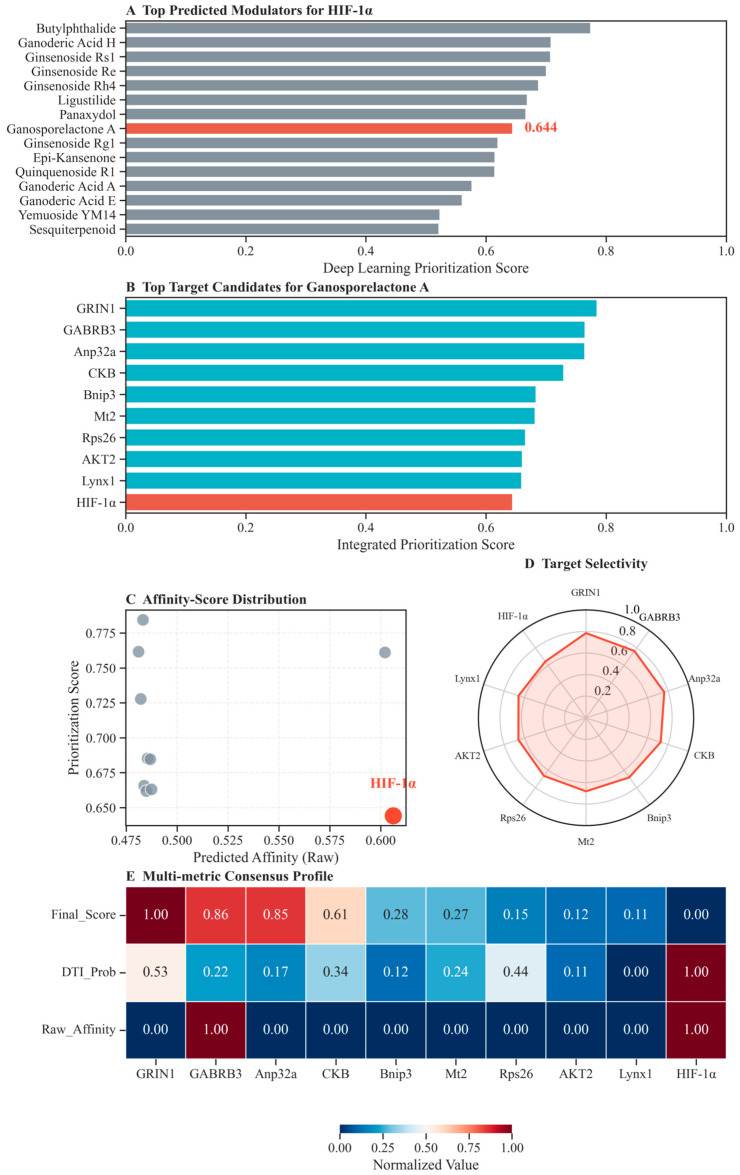
Deep learning-guided prioritization identifies Ganosporelactone A as the specific modulator of the HIF-1α/RAGE axis. (**A**) Compound Prioritization. Vertical bar chart ranking YXYS-derived compounds based on their predicted interaction scores with the core target HIF-1α. Ganosporelactone A (highlighted in red) is identified as the top-ranked modulator. (**B**) Target Profiling. The top predicted targets for Ganosporelactone A. HIF-1α (Score: 0.644) and RAGE (Score: 0.606) are identified as the primary binding partners with the highest integrated scores, consistent with the experimental hypothesis of dual-pathway modulation. (**C**) Affinity-Score Correlation. Scatter plot mapping the predicted binding affinity (Raw Affinity) against the final prioritization score. The HIF-1α interaction (red dot) exhibits both high affinity and high confidence. (**D**) Target Selectivity. Radar chart visualizing the targeting scope of Ganosporelactone A, showing a skewed preference for the HIF-1α and RAGE. (**E**) Multi-Metric Consensus. Heatmap displaying normalized scores across three evaluation metrics, confirming the robustness of the prediction for the identified core targets. Abbreviations: DTIAM, Drug–Target Interaction with Attention Mechanism.

The prioritization analysis revealed a clear hierarchy among the candidate compounds. As shown in Figure 10A, Ganosporelactone A emerged as the top-tier modulator, exhibiting the highest integrated prioritization score for HIF-1α binding among all screened constituents. This result distinguishes Ganosporelactone A as the primary material basis for HIF-1α regulation within the formula. To further elucidate its mechanism of action, we profiled the multi-target spectrum of Ganosporelactone A. Remarkably, the target ranking analysis (Figure 10B) identified HIF-1α (Score: 0.644) and RAGE (Score: 0.606) as its top two high-confidence targets. This finding provides independent computational verification of our “dual-target” hypothesis, suggesting that Ganosporelactone A acts as a synergistic bridge between hypoxic adaptation (HIF-1α) and neuroimmune modulation (RAGE). Additionally, downstream effectors such as VEGF and AKT2 also ranked within the top candidates, indicating the comprehensive modulation of the signaling network.

The robustness of these predictions was corroborated by multi-metric evaluations. The affinity-score correlation analysis (Figure 10C) demonstrated that the HIF-1α interaction pair is characterized by both maximal predicted affinity (A_raw_) and high interaction probability, occupying the optimal upper-right quadrant. Furthermore, the selectivity profile, visualized via a radar chart (Figure 10D) and consensus heatmap (Figure 10E), confirmed that Ganosporelactone A possesses a specific targeting preference for the HIF-1α/RAGE axis compared to peripheral targets.

**Figure 11:**
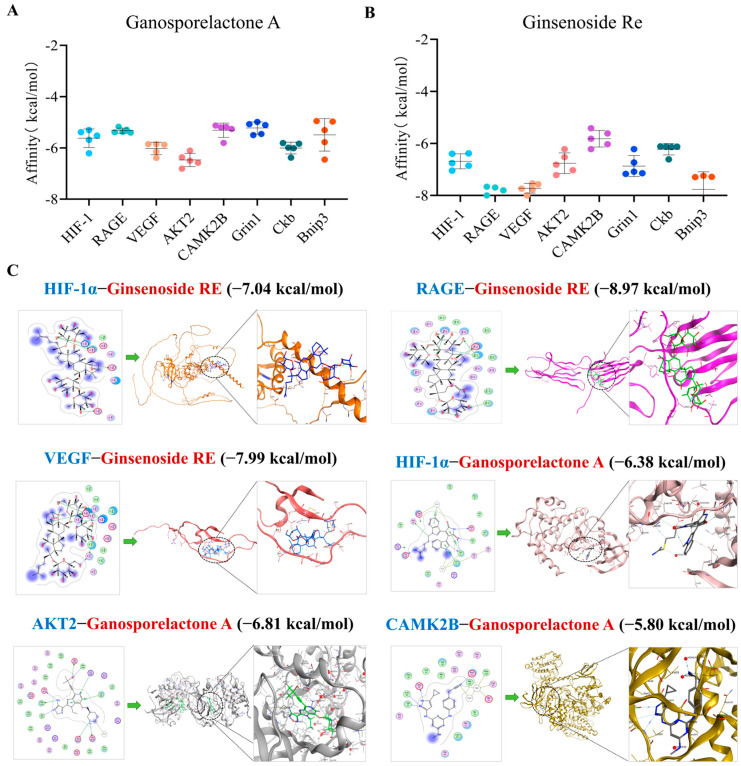
Molecular docking validation of key YXYS constituents with core therapeutic targets. (**A**,**B**) Docking Energy Heatmap. Binding affinity scores (kcal/mol) of Ganosporelactone A and Ginsenoside Re against eight prioritized targets (HIF-1α, RAGE, VEGF, AKT2, CAMK2B, GRIN1, CKB, BNIP3). Darker colors indicate stronger binding affinity. (**C**) Visualization of Binding Interactions. Representative 2D and 3D docking poses of Ganosporelactone A and Ginsenoside Re with HIF-1α and RAGE, highlighting key hydrogen bonds and amino acid residues within the active pockets. The results demonstrate that both compounds can stably bind to the core targets, supporting the multi-component synergistic mechanism of YXYS.

To physically validate these deep learning predictions, we selected eight high-ranking proteins—including the core hubs HIF-1α and RAGE, as well as downstream effectors VEGF, AKT2, CAMK2B, GRIN1, CKB, and BNIP3—for molecular docking. Ginsenoside Re was also included in the docking analysis to evaluate the potential synergistic effects of major YXYS constituents. As shown in Figure 11, flexible molecular docking simulations revealed that both Ganosporelactone A and Ginsenoside Re exhibited strong binding affinities with the catalytic pockets of HIF-1α and RAGE. Specifically, Ganosporelactone A formed stable hydrogen bonds with critical residues, further supporting its selection as the primary candidate for subsequent molecular dynamics simulations.

### 2.13. MD Simulation Results

**Figure 12:**
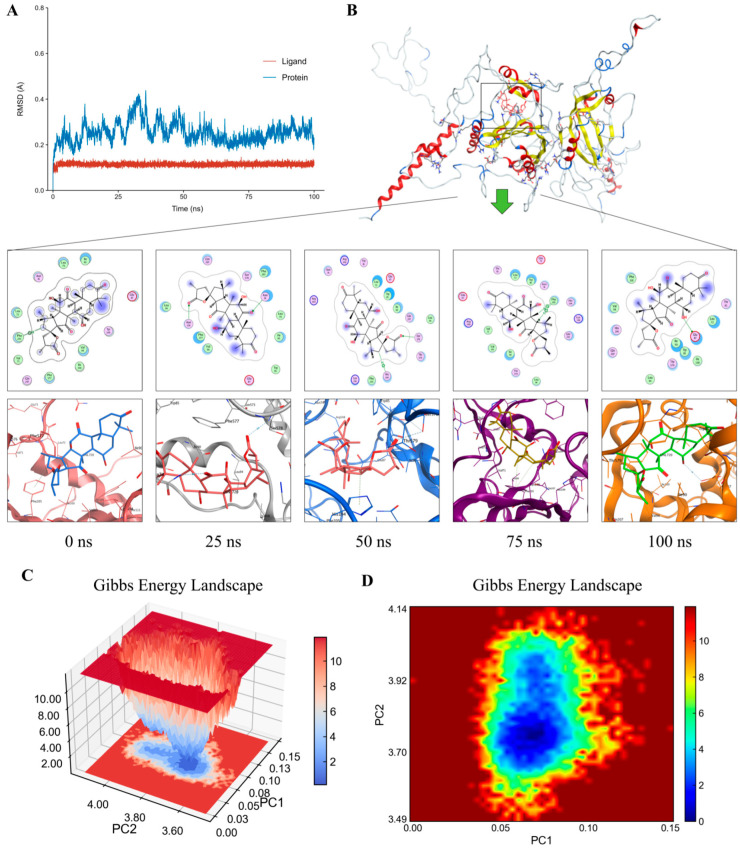
Molecular dynamics (MD) simulation analyses. (**A**) 100-ns MD simulation trajectory of the Ganosporelactone A/HIF-1α complex. The green arrow indicates the extraction and magnified view of the ligand-binding pocket for detailed timeline visualization. The secondary structural elements of the protein are colored to represent α-helices (red), β-sheets (yellow), and loops/coils (blue and gray). (**B**) Conformational evolution and representative binding pose snapshots (0–100 ns) of the Ganosporelactone A/HIF-1α complex. (**C**) Three-dimensional Gibbs free energy landscape and (**D**) corresponding two-dimensional potential of mean force (PMF) projection for unliganded HIF-1α.

To further explore the dynamic behavior of the HIF-1-Ganosporelactone A complex, a 100-nanosecond (ns) molecular dynamics (MD) simulation was conducted to assess the stability of the complex using root mean square deviation (RMSD) and hydrogen bond fluctuations as key parameters (Figure 12A). RMSD, measured in angstroms (Å, 1 Å = 10^−10^ m), quantifies structural deviations over time, providing insight into the stability of the biomolecular complex. A lower RMSD value indicates minimal conformational deviation, suggesting that the ligand–protein complex maintains stability throughout the simulation. The trajectory analysis of the MD simulation revealed that both HIF-1 and Ganosporelactone A exhibited minimal conformational changes between the initial and final states, with no significant deviations observed, indicating that the binding interaction remained stable throughout the 100-ns simulation (Figure 12B). To gain a more detailed understanding of the ligand’s behavior within the binding pocket, structural snapshots of the HIF-1-Ganosporelactone A complex were extracted at specific time points (0, 25,50, 75 and 100 ns). These snapshots demonstrated that Ganosporelactone A consistently remained within the HIF-1 binding domain throughout the simulation, with no signs of dissociation or significant conformational drift. This strong binding stability suggests that Ganosporelactone A may effectively modulate HIF-1 activity, potentially influencing downstream signaling pathways.

The receptor–ligand complex stability was assessed via RMSD, Rg, and Gibbs free energy landscape analyses, yielding 3D free energy surfaces and 2D contour maps (Figure 12C,D). Areas of the landscape with blue and purple hues represent the regions where the complex adopts its most stable conformations, corresponding to the lowest free energy states. In contrast, less stable interactions are indicated by multiple, rough-surfaced energy minima. Strong, stable interactions are characterized by a single, smooth energy minimum. As illustrated, the Gibbs free energy landscape of the HIF-1-Ganosporelactone A complex reveals a distinct, sharp minimum energy zone, signifying a highly stable binding conformation.

### 2.14. Serum Inflammatory Markers and Aβ_1–42_ Levels

**Figure 13:**
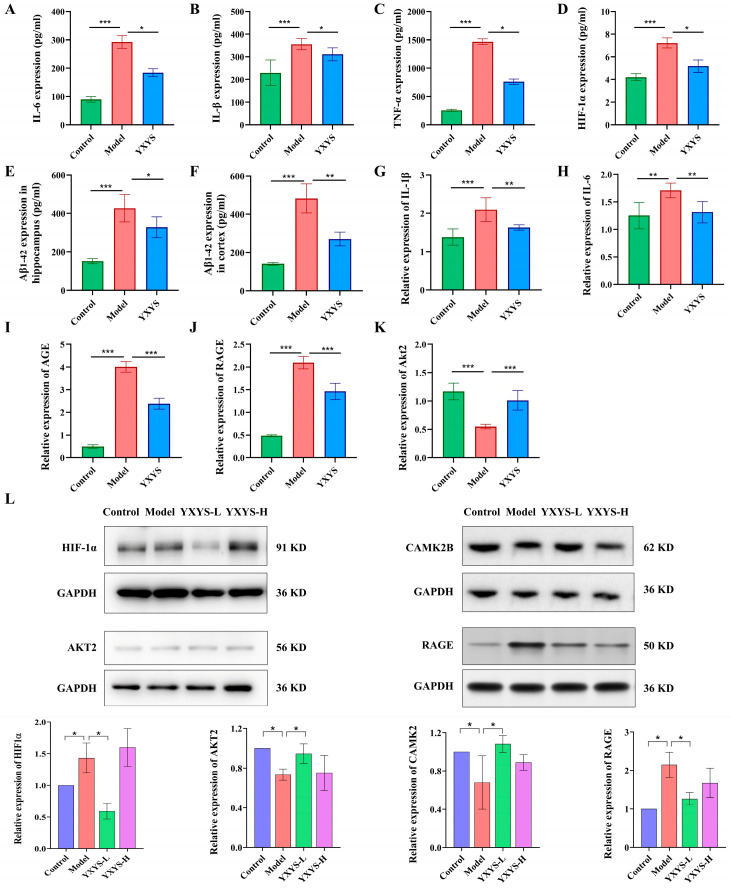
Effects of YXYS on inflammatory, amyloid, and AGE-RAGE pathway markers in AD model mice (*n* = 6). (**A**–**D**) Serum levels of IL-6 (**A**), IL-1β (**B**), TNF-α (**C**), and HIF-1α (**D**). (**E**,**F**) Aβ_1–42_ levels in hippocampus (**E**) and cortex (**F**). (**G**–**K**) Relative mRNA expression of IL-1β (**G**), IL-6 (**H**), AGE (**I**), RAGE (**J**), and AKT2 (**K**) in brain tissues. (**L**) Representative WB and quantification of HIF-1α, AKT2, RAGE and CAMK2B proteins. Data are mean ± SD. * *p* < 0.05, ** *p* < 0.01, *** *p* < 0.001.

Serum levels of pro-inflammatory cytokines (IL-6, IL-1β, TNF-α) and HIF-1α were significantly elevated in the AD model group compared with controls (*p* < 0.001; Figure 13A–D), reflecting systemic inflammation and hypoxia. YXYS treatment (low and high doses) markedly attenuated these elevations (*p* < 0.05), indicating anti-inflammatory and anti-hypoxic effects. Aβ_1–42_ levels in the hippocampus and cortex were substantially increased in the AD model group (*p* < 0.01, Figure 13E,F), consistent with amyloid pathology. HIF-1α in Figure 13L showed a non-linear change across doses (decrease in YXYS-L but a rebound in YXYS-H), and we therefore describe this pattern as a potential dose-window response rather than a strictly dose-dependent effect. YXYS administration significantly lowered Aβ_1–42_ accumulation in both regions (*p* < 0.01), suggesting neuroprotective potential against plaque deposition.

### 2.15. Gene Expression and Protein Levels in AGE-RAGE Pathway

RT-qPCR was conducted to validate the expression profiles of key genes involved in the AGE-RAGE and HIF-1 signaling pathways, with a focus on HIF-1 activation and inflammatory markers in the control, AD model, and YXYS-treated groups. The AD model group exhibited significant upregulation of pro-inflammatory cytokines IL-1β and IL-6 (*p* < 0.001 and *p* < 0.01, Figure 13G,H), which were significantly reduced following YXYS treatment (*p* < 0.01), highlighting its anti-inflammatory effects. AGE expression was significantly higher in the AD model group compared to controls (*p* < 0.001) but was significantly decreased after YXYS treatment (*p* < 0.001, Figure 13I). Likewise, RAGE expression was elevated in the AD model group (*p* < 0.001) and significantly reduced following YXYS administration (*p* < 0.001, Figure 13J). Furthermore, AKT2, a crucial gene in both the AGE-RAGE and HIF-1 pathways, was markedly downregulated in the AD model group (*p* < 0.001) and upregulated after YXYS treatment (*p* < 0.001). These results provide evidence that YXYS modulates key molecular pathways implicated in AD (Figure 13K).

### 2.16. WB Analysis Reveals YXYS-Mediated Modulation of HIF−1 and AGE-RAGE Signaling Pathway Core Proteins

Western blot analysis was conducted to evaluate the expression of key proteins involved in the HIF-1 and AGE-RAGE signaling pathways, including HIF-1α, AKT2, RAGE and CAMK2B. These proteins were selected for their roles in regulating neuroimmune responses and, cellular stress/hypoxia, and pro-survival signaling in AD. As demonstrated in Figure 13L, AD model mice exhibited significant upregulation of HIF-1α and RAGE compared to controls, whereas AKT2 and CAMK2B expressions were markedly downregulated. Notably, YXYS-L treatment reversed these pathological changes, suppressing HIF-1α overexpression (*p* < 0.05) and upregulating AKT2 and CAMK2B expression (*p* < 0.05).

Interestingly, the protein expression pattern observed across the dosage groups was non-monotonic rather than strictly dose-dependent: while the YXYS-L group showed a significant reduction relative to the model, the YXYS-H group exhibited a rebound increase, most noticeably for HIF-1α. This non-linear response is biologically plausible. HIF-1α is a highly dynamic, stress-responsive regulator whose abundance is governed by multi-level control (including rapid post-translational stabilization) and varies with the context and intensity of cellular stress [24]. Moreover, in AD-related settings, the HIF-1 pathway exerts complex and sometimes contradictory roles—potentially protective under certain conditions yet detrimental under others—supporting a biphasic pattern rather than a linear dose-effect relationship [25]. Consistent with this context-dependent regulation, stronger engagement of this pathway does not necessarily confer greater benefit, as hypoxia-induced overactivation of HIF-1α has been associated with enhanced amyloidogenic processing, such as increased BACE1 expression and altered γ-secretase activity [26].

The results collectively suggest that YXYS-L may modulate neuroimmune responses and inflammatory homeostasis, potentially involving HIF-1 and AGE-RAGE pathways, thereby exerting therapeutic effects.

## 3. Discussion

**Figure 14:**
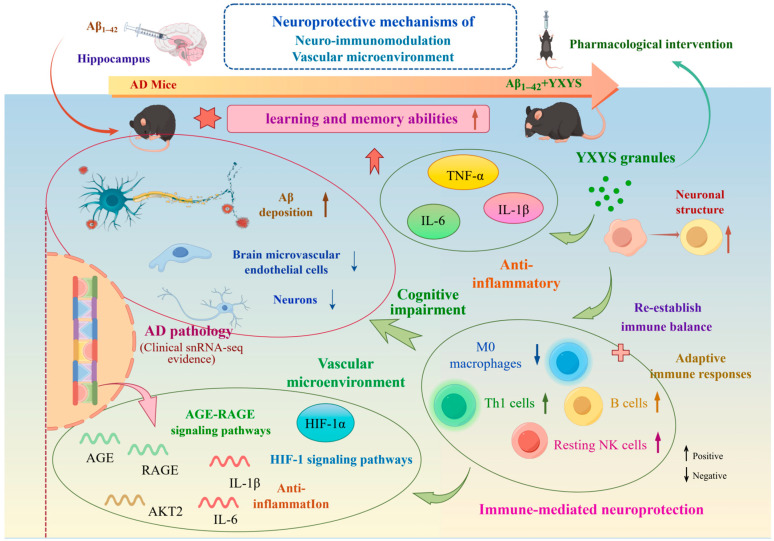
Mechanistic illustration of YXYS therapeutic pathways in the AD model. The small upward (↑) and downward (↓) arrows explicitly indicate the up-regulation (or increase) and down-regulation (or decrease) of specific biomarkers and cell populations, respectively. The directional arrows denote the sequential biological processes and the multi-target pharmacological regulatory pathways of the YXYS intervention. Additionally, distinct colors and spatial groupings are utilized exclusively to visually differentiate functional modules (e.g., neuro-immunomodulation, vascular microenvironment) and specific cellular components, rather than representing quantitative values.

In this study, we integrated multi-omics profiling with computational analyses to explore the potential neuroprotective mechanisms of YXYS in AD. The results indicate that YXYS treatment is associated with improvements in AD-related pathological readouts and cognitive performance, alongside coordinated changes in neuro-immune interactions and the local microenvironment. Pathway analyses highlighted AGE–RAGE and HIF−1 signaling as prominent nodes potentially involved in YXYS-related effects. Consistent with these pathway signatures, YXYS was accompanied by markers suggestive of improved neurovascular homeostasis, reduced neuroinflammatory activation, and enhanced cellular/mitochondrial stress resilience, which may contribute to neuronal preservation and improved cognition in an Aβ-induced AD mouse model. The mechanism diagram (Figure 14) encapsulates YXYS’s multifaceted effects, illustrating its regulation of inflammation, neuroimmune modulation, microenvironmental homeostasis, and neuronal survival.

AD-related pathology often involves significant cellular stress and hypoxia, making the HIF−1 signaling pathway a critical component to investigate [27,28]. Our analysis of human single-nucleus RNA-sequencing data suggested the clinical relevance of this axis, showing reduced vascular and neuronal cell representation in the AD hippocampus together with a transcriptomic pattern consistent with altered HIF−1 signaling and neurovascular dysfunction. These observations informed our subsequent in vivo experiments. In our AD model mice, brain proteomics further supported this association, with differentially expressed proteins enriched in the HIF−1 and VEGF signaling pathways, and HIF−1α appearing as a highly connected node in the protein–protein interaction network. In the treatment group, YXYS was associated with attenuation of the model-related increase in HIF−1α, supported by ELISA, qPCR, and Western blot results. This finding is of interest because sustained or excessive HIF−1α activation has been reported to contribute to blood–brain barrier (BBB) disruption under certain pathological conditions [29]. Further supporting a direct mechanism, our molecular dynamics simulations demonstrated a stable, high-affinity interaction between Ganosporelactone A, a key bioactive compound in YXYS, and HIF−1α over a 100-nanosecond trajectory. This computational evidence provides a direct molecular basis for the formula’s regulatory effect on this critical hypoxia-response pathway, reinforcing its role in stabilizing the microenvironmental homeostasis.

Concurrently, our study highlights the potent immunomodulatory capacity of YXYS. The AGE-RAGE pathway, a primary driver of sterile inflammation and oxidative stress when advanced glycation end-products (AGEs) bind to their receptor (RAGE) [30,31], was consistently identified as a key target across our transcriptomic and proteomic datasets. This pathway is known to contribute to neuroinflammation and vascular injury by sustaining reactive oxygen species production through NF-κB activation [32]. Our results showed that YXYS treatment significantly downregulated the expression of both AGEs and RAGE at the mRNA level, disrupting this pro-inflammatory signaling cascade; consistent with these transcriptional changes, Western blot analysis further confirmed a marked reduction in RAGE protein expression after YXYS treatment. This molecular suppression translated into tangible functional outcomes. At the systemic level, ELISA results confirmed that YXYS significantly reduced serum levels of pro-inflammatory cytokines IL-6, IL-1β, and TNF-α. At the central level, immune cell infiltration analysis revealed that YXYS rebalanced the neuroimmune landscape, reversing the pathological increase in pro-inflammatory M0 macrophages and neutrophils while promoting a more regulated environment characterized by increased Th1 cells, memory B cells, and resting NK cell populations. These findings underscore that YXYS exerts robust immunomodulatory effects in AD, attenuating neuroinflammation, rebalancing immune cell dynamics, and thereby supporting neuroprotection.

The multi-target effects of YXYS extend beyond the tissue level to promoting cellular and mitochondrial resilience. Using Aβ_25–35_-challenged HT22 cells to model AD-related neuronal stress, we found that YXYS treatment provided robust cytoprotection by significantly restoring mitochondrial membrane potential, reducing intracellular ROS production, and upregulating NRF1 and TOM20—key regulators of mitochondrial biogenesis and function. These in vitro findings were powerfully corroborated by our integrated multi-omics analysis in vivo. From 293 overlapping targets identified between transcriptomic and proteomic datasets, O2PLS analysis highlighted the mitochondrial protein Mtnd2 and the chaperone Hsp70 as key modulators of YXYS’s effects. This convergence of evidence from cellular models to systems-level omics reinforces the concept that YXYS confers neuroprotection by reinforcing fundamental cellular bioenergetics and stress-response pathways. These molecular and cellular improvements provided the foundation for observed functional recovery. In both the MWM and NOR tests, YXYS-treated mice exhibited significantly improved cognitive performance. This behavioral restoration was directly correlated with preserved hippocampal architecture, as evidenced by reduced vacuolation in HE staining and a significant increase in Nissl-positive neuronal density, particularly in the dentate gyrus, which surpassed the effects of the positive control drug, donepezil.

Crucially, our multi-omics network analysis identified AKT2 as a pivotal cross-talk node bridging the AGE-RAGE and HIF−1 signaling axes [33,34,35]. The PI3K/AKT pathway is fundamental for neuronal survival, and its impairment is a hallmark of AD pathology, contributing to both vascular compromise and apoptotic cell death [36]. In our model, the downregulation of AKT2 signaled compromised neuronal survival [37], whereas YXYS treatment effectively restored AKT2 expression at both mRNA and protein levels. This restoration is particularly significant because AKT2 activation can simultaneously inhibit AGE-induced pro-inflammatory signaling and modulate HIF−1−mediated hypoxic responses, thereby underscoring the formula’s ability to engage multiple pathways synergistically to enhance cell survival.

Identifying the specific active components from a complex herbal formula remains a challenge. While our structure-based molecular docking demonstrated that Ginsenoside Re fits precisely into the catalytic pockets of VEGF and HIF−1α, our newly introduced Deep Learning analysis provided a complementary perspective based on global chemical semantic features. Through self-supervised learning, DTIAM prioritized Ganosporelactone A as the top-tier modulator, exhibiting the highest interaction probability for HIF−1α (Score: 0.644) and RAGE (Score: 0.606) among all screened compounds. Notably, Ganosporelactone A also showed strong predicted affinity for downstream effectors, including AKT2 (Score: 0.661), VEGF (Score: 0.612), and CAMK2G (Score: 0.561). This discrepancy between docking (structural fit) and deep learning (feature representation) likely reflects the multi-component, multi-target synergy inherent in YXYS. We propose that Ginsenoside Re may act as a ‘specific locker’ for downstream effectors, whereas Ganosporelactone A serves as a ‘broad-spectrum anchor’ regulating the upstream HIF−1α/RAGE axis. This dual-validation approach—combining physical simulation with AI-driven prioritization—significantly enhances the reliability of our material basis screening. This multi-target action on the core signaling network was definitively confirmed by Western blot analysis, which validated the regulatory effects of YXYS on HIF−1α, AKT2, RAGE, and CAMK2B protein levels.

This study has several limitations. First, while we profiled the major constituents of YXYS and prioritized putative compound–target associations through computational analyses, the complex synergistic interactions among multiple constituents remain insufficiently characterized, and the full polypharmacological profile may therefore not be completely captured. Second, we did not perform post-dose serum or brain exposure profiling to confirm which prototype constituents and/or metabolites are present in vivo, nor did we evaluate blood–brain barrier (BBB) permeability. Accordingly, the targets and pathways proposed here are inferred from the extract composition and should be regarded as hypothesis-generating, pending validation with in vivo exposure–guided evidence. Finally, systematic characterization of pharmacokinetics and bioavailability of candidate active components will be important in future work to inform dosing strategies and improve the clinical translatability of these findings. Our study focused primarily on Aβ-driven pathology; future research should also explore the effects of YXYS on tauopathy and synaptic plasticity to fully delineate its therapeutic scope. Ultimately, despite the promising preclinical evidence presented here, randomized controlled clinical trials are essential to validate the efficacy and safety of YXYS in human AD patients.

In conclusion, our study provides a robust, multi-layered mechanistic framework for the therapeutic action of YXYS in Alzheimer’s disease. By targeting the critical AGE-RAGE and HIF−1 signaling pathways, YXYS reestablishes neuro-immune homeostasis and mitigates AD-related pathology. It mitigates neuroinflammation by reducing pro-inflammatory cytokine production and rebalancing immune cell populations; it alleviates hypoxic stress by modulating the HIF−1α pathway; and it promotes neuronal resilience by restoring mitochondrial function and activating pro-survival signaling through molecules like AKT2. These findings support the hypothesis that YXYS may act through multi-target network regulation.

## 4. Materials and Methods

### 4.1. Single-Nucleus RNA Sequencing Analysis of Human AD Hippocampus

#### 4.1.1. Data Acquisition

Single-nucleus RNA sequencing (snRNA-seq) data were obtained from the Gene Expression Omnibus (GEO) database under accession number GSE163577. Analysis focused on hippocampus-derived samples from postmortem human tissue of individuals with AD (AD; *n* = 3) and no cognitive impairment (NCI) controls (*n* = 3), generated via a custom vessel isolation and nuclei extraction protocol (VINE-seq) [38].

#### 4.1.2. Data Pre-Processing, Quality Control, and Integration

All analyses were performed using the R package Seurat (version 5). Genes detected in fewer than 3 nuclei were excluded. Nuclei were filtered based on quality metrics, retaining those with 200–5000 unique features and mitochondrial gene content below 10% to eliminate low-quality or apoptotic nuclei. Quality control metrics, including distributions of nCount_RNA, nFeature_RNA, percent.mt, and percent.rb, are visualized in violin plots, confirming comparable profiles across AD and NCI samples.

Following quality control, normalization was applied using the NormalizeData function (“LogNormalize” method, scale factor = 10,000). The 3000 most highly variable genes were selected with FindVariableFeatures (vst method).

#### 4.1.3. Dimensionality Reduction, Clustering, and Cell Type Annotation

Principal component analysis (PCA) embeddings were used for downstream processing. The first 12 principal components were selected based on elbow plot and DimHeatmap assessments. Clustering was performed with FindNeighbors and FindClusters (resolution = 0.12) for optimal cluster resolution. Uniform manifold approximation and projection (UMAP) was employed for visualization.

Cell types were annotated manually using canonical marker genes from prior literature. Cluster-specific markers were identified via FindAllMarkers (Wilcoxon rank-sum test), with significance defined as adjusted *p* < 0.05 and log_2_(fold change) > 0.25.

#### 4.1.4. Differential Expression and Functional Enrichment Analysis

Group-wise differential expression between AD and NCI was evaluated within each cell type using the Wilcoxon rank-sum test.

Differentially expressed genes (DEGs) underwent functional enrichment with the clusterProfiler R package, including Gene Ontology (GO) terms (Biological Process, Molecular Function, Cellular Component) and Kyoto Encyclopedia of Genes and Genomes (KEGG) pathways. Enrichment significance was set at *p* < 0.05.

#### 4.1.5. Cell Proportion Analysis

Cell type proportions were calculated per sample. Differences between AD and NCI groups were tested using the Wilcoxon rank-sum test.

### 4.2. UPLC-QTOF-MS Analysis of Bioactive Compounds

#### 4.2.1. Drug and Sample Preparation

The YXYS granules were provided by Guangdong Yifang Pharmaceutical Co., Ltd. (Guangzhou, China, Lot No. 353980). The formulation comprises six herbal components: *Panax quinquefolius* L., *Panax notoginseng* (Burkill) F.H. Chen, *Ganoderma lucidum* (Leyss. ex Fr.) Karst, *Cornus officinalis* Sieb. et Zucc., *Artemisia anomala* S. Moore, and *Ligusticum chuanxiong* Hort. The components are combined in a fixed ratio of 3:3:5:10:10:5.

YXYS powder (200 mg) was subjected to ultrasonic extraction using 1 mL of 50% methanol-water solution (*v*/*v*) for 30 min. The resulting extract was passed through a 0.22 μm membrane filter and subsequently transferred into vials for instrumental analysis.

#### 4.2.2. UPLC-QTOF-MS Analysis

UPLC–QTOF–MS analysis was performed on an ACQUITY UPLC H-Class system coupled to a Xevo G2-XS QTof mass spectrometer (Waters, Milford, MA, USA). Both positive and negative electrospray ionization (ESI) modes were employed. The capillary voltage was set at +3.0 kV in positive mode and −2.5 kV in negative mode. The sample cone voltage was 40 V, and the source offset voltage was 20–80 V. The source temperature was maintained at 120 °C, and the desolvation temperature was set to 400 °C. Cone gas and desolvation gas flows were set to 50 L/h and 800 L/h, respectively, with a nebulizer pressure of 6.0 bar.

Chromatographic separation was achieved on an ACQUITY UPLC BEH C18 column (2.1 × 100 mm, 1.8 μm; Waters, Milford, MA, USA) maintained at 35 °C. The mobile phase consisted of (A) deionized water containing 0.1% formic acid and (B) acetonitrile containing 0.1% formic acid. The flow rate was 0.3 mL/min and the injection volume was 10 μL. A 45-min gradient elution program was applied as follows: 0 min, 100% A/0% B; 10 min, 70% A/30% B; 25 min, 60% A/40% B; 30 min, 50% A/50% B; 40 min, 30% A/70% B; and 45 min, 0% A/100% B. To verify compound identification, reference standards were co-injected with the samples. This method generated a comprehensive chromatographic and mass spectral profile of the YXYS extract for subsequent compound characterization.

#### 4.2.3. Data Processing

Data acquisition and preliminary analysis were performed using MassLynx V4.2, where a dedicated processing method was established for peak extraction and MS data interpretation. Compound searching and assisted identification were conducted on the UNIFI platform by querying the built-in Traditional Medicine Library (TML). In MassLynx V4.2, the data-processing method was set with a mass error tolerance of ±10 ppm and a response threshold of >3000, and compounds lacking high-energy fragment ions were excluded. Putative identifications were then achieved by comparing the quasi-molecular ion peaks and MS/MS fragmentation patterns with those recorded in the database [39].

### 4.3. Network Pharmacological Analysis

#### 4.3.1. Potential Targets Identification

Based on the UPLC-QTOF-MS analysis of the YXYS extract, a total of 31 chemical constituents were identified and structurally characterized. These experimentally verified compounds served as the basis for subsequent target prediction. Candidate target genes were predicted using both SuperPred (https://prediction.charite.de/, accessed on 6 August 2025) and Swiss Target Prediction (http://www.swisstargetprediction.ch/, accessed on 6 August 2025), and gene identifiers were standardized using the UniProt database (https://www.uniprot.org/, accessed on 6 August 2025).

#### 4.3.2. Network Construction

The AD gene data was retrieved from the DisGeNET database (https://www.disgenet.org/search, accessed on 7 August 2025). Using the Venny 2.1 online tool (https://bioinfogp.cnb.csic.es/tools/venny/index.html, accessed on 7 August 2025), the genes common between the predicted YXYS-related targets and those associated with AD were identified. These overlapping targets were then subjected to functional enrichment analysis, including Gene Ontology (GO) and Kyoto Encyclopedia of Genes and Genomes (KEGG) pathway analyses. A comprehensive network illustrating the relationships between drug, disease, target, and pathway was constructed and visualized using Cytoscape software (version 3.9.1).

### 4.4. Cell Experiments

#### 4.4.1. Preparation of YXYS-Containing Serum

Five healthy adult Sprague–Dawley rats (200–220 g) were administered YXYS extract (3.32 g/kg/day) for 3 consecutive days. Control animals (*n* = 5) received equivalent volumes of sterile saline. At 1 h following the final administration, venous blood was collected under anesthesia via orbital enucleation. After coagulation (30 min, 4 °C), the samples were centrifuged (3000× *g*, 15 min, 4 °C) to isolate the serum. The complement proteins in the serum were inactivated by heat treatment (56 °C, 30 min), followed by sterile filtration through a 0.22 μm membrane.

#### 4.4.2. Cell Viability Assay

The immortalized mouse hippocampal cell line HT22 was obtained from the Cell Resource Center, Institute of the Chinese Academy of Sciences (Shanghai, China). The immortalized mouse hippocampal cell line HT22 was obtained from the Cell Resource Center, Institute of the Chinese Academy of Sciences (Shanghai, China). HT22 mouse hippocampal neuronal cells were maintained in DMEM supplemented with 10% fetal bovine serum (FBS) and 1% penicillin/streptomycin at 37 °C in a humidified incubator with 5% CO_2_. Aβ_25–35_ peptide was prepared according to the kit instructions: Aβ_25–35_ was reconstituted in sterile saline to 1 mM and incubated (aged) at 37 °C for 7 days. Prior to use, the peptide solution was diluted with complete medium to the desired working concentrations. The selection of the Aβ_25–35_ peptide, rather than full-length Aβ_1–40_ or Aβ_1–42_, to induce cytotoxicity was based on its established pharmacological properties and exceptional sequence conservation. Aβ_25–35_ represents the biologically active, core toxic fragment of the full-length peptide, possessing the capability to spontaneously and rapidly self-assemble into β-sheet structures and highly toxic oligomers in aqueous solutions [40]. This provides a highly stable and reproducible acute neurotoxicity model optimal for in vitro screening. Furthermore, sequence alignment confirms that while the N-terminal region of Aβ differs slightly between humans and mice (e.g., R5G, Y10F, H13R substitutions), the 25–35 fragment (GSNKGAIIGLM) is 100% identical and evolutionarily conserved between the two species [41]. Consequently, utilizing this synthetic peptide ensures perfect sequence homology with the native receptors of the murine HT22 cell line.

For the cytotoxicity assay, logarithmic-phase HT22 cells were seeded into 96-well plates at 5 × 10^4^ cells/mL (100 μL per well) and allowed to adhere for 24 h. After removal of the medium, cells were treated with Aβ_25–35_ at different concentrations (5, 10, 20, 40, and 80 μM) for 24 h to determine the optimal modeling concentration. Cells in the control group received vehicle/blank serum.

For the drug-protection assay, HT22 cells were seeded as described above and, after 24 h of attachment, were pretreated with different concentrations of medicated serum (5%, 10%, 15%, and 20%, *v*/*v*) for 24 h. Subsequently, Aβ_25–35_ was added in the presence of YXYS-medicated serum to a final concentration of 20 μM, followed by further incubation for 24 h. Cells in the control group were treated with the corresponding concentrations of blank serum. At the end of treatment, cell viability was assessed using the CCK-8 assay. Briefly, 10 μL of CCK-8 reagent was added to each well, followed by incubation at 37 °C for 1 h. Absorbance was measured at 450 nm, and cell viability was calculated accordingly.

#### 4.4.3. Mitochondrial Membrane Potential (MMP) Assay

The mitochondrial membrane potential (MMP) was assessed using the JC-1 fluorescent probe (Beyotime, C2003S). JC-1 aggregates in healthy mitochondria, producing red fluorescence, whereas in depolarized mitochondria, it remains as green fluorescent monomers. The ratio of red to green fluorescence was used to determine the mitochondrial polarization. HT22 cells were treated with the JC-1 staining solution at 37 °C for 20 min, followed by washing with PBS three times. Fluorescence images were obtained using an inverted fluorescence microscope (OLYMPUS IX53, Tokyo, Japan).

#### 4.4.4. Intracellular Reactive Oxygen Species (ROS) Detection

ROS levels were assessed in HT22 cells using the DCFH-DA fluorescent probe (Nanjing Jiancheng Bioengineering Institute, Nanjing, China, E004-1-1). Cells were seeded in six-well plates and subjected to the appropriate treatments. After treatment, the cells were rinsed with PBS and incubated with 10 µM DCFH-DA at 37 °C for 20 min in a 5% CO_2_ environment. After incubation, excess dye was removed by washing with PBS three times, and the fluorescence was captured using an inverted fluorescence microscope (OLYMPUS IX53, Tokyo, Japan).

#### 4.4.5. Immunofluorescence Staining of NRF1 and TOM20

For immunocytochemistry, HT22 cells were cultured on glass coverslips. Following treatment, cells were fixed with 4% paraformaldehyde for 15 min, permeabilized with 0.1% Triton X-100 for 10 min, and blocked with 5% bovine serum albumin (BSA) for 1 h at room temperature. The cells were incubated overnight at 4 °C with primary antibodies against NRF1 (Proteintech, Rosemont, IL, USA, 12381-1-AP) and TOM20 (Abcam, Cambridge, UK, ab186735). After washing with PBS, Alexa Fluor 488-conjugated secondary antibodies (Abcam) were applied for 1 h at room temperature. Nuclear staining was done with DAPI (Beyotime, Shanghai, China, C1005), and images were obtained using a confocal fluorescence microscope (OLYMPUS IX53, Tokyo, Japan).

### 4.5. Experimental Animals and AD Model Induction

Forty-eight male C57BL/6J mice (SPF grade), each weighing approximately 15 ± 2 g, were obtained from GemPharmatech Co., Ltd. (Beijing, China; Permit No. SCXK [Beijing] 2023-0008). All animals were maintained in a specific pathogen-free (SPF) facility at the Institute of Basic Theory of Chinese Medicine, affiliated with the China Academy of Chinese Medical Sciences (Permit No. SYXK [Beijing] 2016-0021). The housing environment was stringently regulated, maintaining a stable temperature of 23 ± 2 °C, relative humidity of 50 ± 10%, and a 12-h light/dark photoperiod. All procedures involving animals were performed in strict accordance with the Guidelines for Ethical Review of Laboratory Animal Welfare (GB/T 35892-2018). The experimental protocol was reviewed and approved by the ethics committee of Beijing Morningrise Hi-Tech Biotechnology Co., Ltd. (Beijing, China; Approval No. 20240008MSE-2).

Following a 3-day acclimation period, 48 mice were divided into two groups: sham surgery group (*n* = 12) and AD model group (*n* = 36). On day 8, the AD model group mice were randomly subdivided into the following groups: Model, Donepezil (3 mg/kg), YXYS-L (4.68 g/kg, clinical equivalent via body surface area scaling) and YXYS-H (9.36 g/kg, 2× clinical dose). The model group was induced with AD through the bilateral injection of Aβ_1–42_ into the intracerebroventricular space [42]. Amyloid β peptide (1–42) was obtained from Meilunbio (Liaoning, China, CAS: 166090-74-0). Donepezil hydrochloride was sourced from Eisai Pharmaceutical Co., Ltd. (Suzhou, China, Lot No. H20070181). The sham surgery and model groups received equivalent volumes of distilled water. All treatment groups received their respective treatments via oral gavage at 0.1 mL/10 g body weight daily for 30 consecutive days.

#### 4.5.1. Novel Object Recognition (NOR) Test

The NOR test was conducted in three sequential stages: habituation, familiarization, and testing. In the familiarization phase, two identical objects were placed at fixed positions within the testing arena, each located 10 cm away from the side and back walls. Mice were permitted to explore the environment freely for 5 min. After a 24-h interval, the testing phase was carried out by replacing one of the previously introduced objects with a new one that differed in shape and color but was comparable in size. Mice were again allowed unrestricted exploration of the arena for 5 min.

Exploratory behaviors, including the number of interactions and the duration spent investigating each object, were recorded using an automated video tracking system (Shanghai Xinyuan Information Technology Co., Ltd., Shanghai, China). The location preference index was computed as:

(time spent on left or right object/total exploration time) × 100%

and the discrimination index was calculated as:

(time spent on the novel object/total exploration time) × 100%.

#### 4.5.2. Passive Avoidance Test

The passive avoidance experiment was performed using a light–dark shuttle box equipped with copper grid flooring in both compartments, connected to a regulated power source. The dark chamber delivered a 1 mA, 50 Hz alternating current shock for 3 s. A circular hole (3 cm in diameter) linked the two compartments, allowing free movement between them. Mice were initially allowed a 5-min adaptation period in the apparatus. After habituation, each mouse was placed in the illuminated chamber while the power supply to the dark chamber was activated. Driven by their natural tendency to prefer dark environments, the mice entered the dark compartment, received an electric shock, and subsequently returned to the light chamber, thereby associating the dark compartment with the aversive stimulus. This training protocol was repeated. After 24 h, the retention test was conducted. The following parameters were measured: latency to enter the dark chamber (time from trial initiation to shock), the number of errors (shocks received within 5 min), and the duration spent in the light compartment. To minimize experimental interference, the apparatus was disinfected with 70% ethanol between each trial.

#### 4.5.3. Morris Water Maze (MWM)

The Morris water maze assessed spatial learning and memory using a 120 cm circular pool with opaque water. The pool was partitioned into four quadrants, each identified by distinct visual symbols affixed to the surrounding walls.

A visible platform trial was conducted on the first day to allow for acclimation. Hidden platform training followed on days 2 through 4. Each trial allowed mice to explore the pool for up to 60 s. If the platform was found, the animal remained on it for 5 s; otherwise, it was gently directed to the platform and kept there for 15 s. Each mouse underwent four training sessions per day.

On day 5, a probe trial tested spatial memory retention without the platform. Search patterns and swimming paths were recorded and analyzed via an automated tracking system.

### 4.6. Tissue Collection

Following completion of behavioral testing, mice were euthanized for biological sampling. Blood was collected via enucleation, followed by transcardial perfusion with normal saline. Three mice per group were randomly selected for anesthesia and perfused with saline only. Their brains were excised and hemisected; one hemisphere was stored for RNA sequencing and quantitative PCR, while the other underwent fixation. In a separate set, three additional mice were perfused with saline and PFA. The right hemisphere was cryosectioned, while the left was paraffin-embedded for HE and Nissl staining. For transcriptomic analyses, the brain tissues were carefully isolated and snap-frozen at −80 °C. The remaining animals were perfused with 4% PFA, and their brain and peripheral tissues were collected and stored in the same fixative.

### 4.7. HE and Nissl Staining

To evaluate neuronal integrity and survival, haematoxylin–eosin (HE) and Nissl staining were conducted on brain sections. Following behavioral testing, brains were collected, post-fixed in 4% paraformaldehyde, and subjected to dehydration using a graded ethanol series before being embedded in paraffin. Coronal slices of 5 μm thickness were obtained using a rotary microtome. For HE staining, sections were deparaffinized, rehydrated, stained with hematoxylin (5 min), and counterstained with eosin (2 min). For Nissl staining, brain slices were incubated with 0.1% cresyl violet solution at room temperature for 10 min to label Nissl bodies. All stained sections were examined under light microscope.

Quantification of Nissl-positive neurons was performed using ImageJ software (version 1.53) in selected brain regions including the cortex, CA1, CA3, and DG of the hippocampus. Results were presented as the average number of positively stained neurons per section.

### 4.8. Transcriptomic Analysis

#### 4.8.1. Quality Control of Sequencing Data

RNA sequencing was performed in the control, model, and YXYS-treated groups. Three mice were randomly selected from each group as biological replicates (*n* = 3). The hippocampus from each mouse was collected as an independent sample for total RNA extraction, with no pooling between animals. RNA concentration and purity were measured by NanoDrop^®^, and integrity by the Agilent RNA Nano 6000 Assay. cDNA libraries were prepared with the NEBNext^®^ Ultra Kit, amplified by PCR, and validated via Agilent 2100 and RT-qPCR. High-quality reads (Q20/Q30, GC content) were filtered and aligned to the reference genome using HISAT2 for transcriptomic analysis.

#### 4.8.2. Analysis of Differential Gene Expression

Raw sequencing reads were aligned to the reference genome using HISAT2. Subsequently, featureCounts was employed to quantify the read counts for each gene. The raw count matrix was directly used as input for DESeq2 (R package) to perform differential expression analysis, as this method models the negative binomial distribution of raw counts. Genes with an adjusted *p*-value (FDR) < 0.05 and |log2FoldChange| > 1 were identified as Differentially Expressed Genes (DEGs). FPKM (Fragments Per Kilobase of transcript per Million mapped reads) values were calculated solely for data visualization purposes.

#### 4.8.3. Gene Set Enrichment Analysis (GSEA)

GSEA was performed using the GSEA tool (http://www.broadinstitute.org/gsea/index.jsp, accessed on 6 August 2025). This analysis targeted KEGG datasets enriched with DEGs derived from the experimental groups, including those treated with YXYS. Enrichment scores were calculated, and significance levels were evaluated with multiple hypothesis testing corrections. The OmicShare tool was used to generate heatmaps, showcasing fold-change data for DEGs enriched in common signaling pathways across all study groups.

#### 4.8.4. Evaluation of Immune Cell Infiltration

To strictly assess the immune landscape in mouse brain tissue, we employed ImmuCC, a computational framework specifically designed for deconvolution of mouse transcriptomic data. Unlike human-based methods, ImmuCC utilizes a validated mouse-specific leukocyte signature matrix to accurately infer the relative proportions of 25 immune cell types, including Microglia, Macrophages, and T cells. The analysis was performed with 1000 permutations, and samples with a *p*-value < 0.05 were retained for downstream comparison between the AD model and YXYS-treated groups.

#### 4.8.5. Proteomics Analysis

Protein concentration was measured at 280 nm using NanoDrop. The FASP protocol facilitated enzymatic digestion and detergent removal via YM-30 filters. Samples were washed with urea and ammonium bicarbonate, alkylated with iodoacetamide, and digested overnight with trypsin at a 100:1 ratio. Peptides were collected by centrifugation. The peptides were analyzed using an Orbitrap Fusion Lumos Tribrid mass spectrometer (Thermo Fisher Scientific, Waltham, MA, USA) coupled with an EASY-nLC 1000 nanoflow HPLC system and a C18 reversed-phase column (Welch Materials, Shanghai, China; 10 cm length, 75 µm inner diameter). Peptide separation was achieved by applying a 75-min linear gradient, ranging from 3% to 100% buffer B (99.5% acetonitrile, 0.5% formic acid). The full LC-MS/MS procedure, including sample loading and system washing, took 90 min.

Electrospray ionization was conducted at a voltage of 2.0 kV. During MS/MS data acquisition, a dynamic exclusion time of 18 s was applied to reduce redundancy. MS1 spectra were recorded at a resolution of 70,000 with an automatic gain control (AGC) target of 3 × 10^6^ and a maximum injection time of 20 ms. For MS2 scans, a resolution of 17,500 was used, with an AGC target of 1 × 10^6^ and a maximum injection time of 60 ms. The scan range was set to 300–1400 m/z, and the top 20 precursor ions were selected for fragmentation and further analysis.

The raw data from mass spectrometry were processed by searching against databases to identify peptide sequences and proteins. Statistical analysis of differentially expressed proteins (DEPs) was performed in RStudio 2025.05.1 ("Mariposa Orchid" Release), with significance defined by a FDR threshold of <0.05 when comparing the control, model, and treatment groups. Subsequent analyses focused on exploring the functional implications and pathway associations of these DEPs.

### 4.9. Multi-Omics Integrative Analysis of Transcriptomic and Proteomic Data

#### 4.9.1. Transcriptomic and Proteomic Data Acquisition

Transcriptomic data were derived from RNA sequencing (RNA-seq) analyses, and proteomic data were obtained from LC-MS/MS analyses. Both datasets were generated from comparable biological samples to ensure consistency across omics layers. Quality control (QC) procedures were applied to filter out low-quality reads from the transcriptomic data and proteins with low spectral counts from the proteomic data. Normalization was performed to account for technical variations, and log transformation was applied to both RNA and protein expression levels.

#### 4.9.2. Data Integration and Alignment

Gene and protein identifiers from transcriptomic and proteomic datasets were aligned based on their respective IDs. In cases of non-matching IDs, a cross-referencing table was used to establish correspondence. Only overlapping genes/proteins from both datasets were included in the integrative analysis, ensuring a focus on the features shared between the transcriptomic and proteomic layers.

#### 4.9.3. Differential Expression Profiling Analysis

Differential expression analysis was independently conducted for both RNA and protein datasets, using FC thresholds of 2-fold for RNA (log_2_FC > 1 or < −1) and 1.2-fold for proteins (log_2_FC > 0.263 or < −0.263). Significance was assessed using adjusted *p*-values (e.g., false discovery rate [FDR] < 0.05), and genes/proteins meeting these criteria were considered differentially expressed.

### 4.10. ELISA Analysis of Serum and Brain Tissues

Enzyme-linked immunosorbent assay (ELISA) kits were used to measure pro-inflammatory cytokines (IL−6, IL−1β, TNF-α), HIF−1α in serum, and Aβ_1–42_ levels in brain tissues. Kits for IL−6 (CB10187-Mu), IL−1β (CB10173−Mu), TNF-α (CB10851−Mu), HIF−1α (serum) (CB10263-Mu), and Aβ_1−42_ (brain) (CB10126−Mu) were from Coibo Bio, Shanghai, China.

### 4.11. Quantitative Reverse Transcription Polymerase Chain Reaction (qRT-PCR)

Total RNA was extracted from hippocampal tissue using the nucleic acid extraction reagent (batch no. Ehan Xiebei 20170012) from Wuhan Zhongzhi Biotechnology Co., Ltd., Wuhan, China. RNA concentration and purity were assessed with the Jiapeng Nano-600 micro-volume nucleic acid analyzer (Shanghai Jiapeng Technology Co., Ltd., Shanghai, China), and integrity was verified by electrophoresis on a 1% agarose gel prepared with agarose (batch no.: HZ0191) from Shanghai Huzhen Biotechnology Co., Ltd. (Shanghai, China). Subsequently, 1 μg of RNA was reverse transcribed using the PrimeRT series gDNA removal first-strand cDNA synthesis master premix (batch no.: RT-01032) from Foregene Biotech Co., Ltd., Chengdu, China. Quantitative real-time PCR (qRT-PCR) was performed using SuperReal PreMix Plus (SYBR Green) premix (cat. no.: FP205) from TIANGEN Biotech (Beijing) Co., Ltd. (Beijing, China) on the HRA-9600 Real-Time Fluorescent Quantitative PCR System from Hongren Bio. Gene-specific primers are listed in Table 2.

The thermal cycling conditions were: initial denaturation at 95 °C for 5 min, followed by 40 cycles of denaturation at 95 °C for 15 s and annealing/extension at 60 °C for 30 s, with fluorescence acquisition at 60 °C. GAPDH was used as the internal reference gene, and relative mRNA expression levels were calculated using the 2^−ΔΔCt^ method after confirming comparable amplification efficiencies between target and reference assays. Each sample was analyzed in technical triplicate.

The thermal cycling program consisted of an initial denaturation at 95 °C for 5 min, followed by 40 cycles of 95 °C for 15 s and 60 °C for 30 s. GAPDH served as the reference gene, and relative gene expression was calculated using the 2^−ΔΔCt^ method. All reactions were performed in triplicate.

### 4.12. Western Blotting (WB) Analysis

Proteins were extracted from brain tissues using RIPA buffer supplemented with a protease inhibitor cocktail (Beyotime, Shanghai, China), and concentrations were determined using a BCA assay kit (Beyotime). Equal amounts of protein were separated by 12% SDS-PAGE and electrotransferred onto PVDF membranes (Millipore, Beijing, China) using a semi-dry transfer method for 50 min. For RAGE and AKT2, the Thermo Scientific PageRuler^TM^ Prestained Protein Ladder (Cat# 26616; 10–180 kDa: 10, 15, 25, 35, 40, 55, 70, 100, 130, 180 kDa) was used, and for CAMK2β and HIF−1α, the PageRuler^TM^ Plus Prestained Protein Ladder (Cat# 26619; 10–250 kDa: 10, 15, 25, 35, 55, 70, 100, 130, 250 kDa). Membranes were blocked with TBST buffer (20 mM Tris, 500 mM NaCl, 0.05% Tween-20) containing 5% non-fat dry milk (BD Biosciences, Shanghai, China) for 1 h at room temperature, then incubated overnight at 4 °C with primary antibodies: rabbit monoclonal anti-HIF−1α (recombinant mAb, lot: BD09097733), rabbit polyclonal anti-RAGE (lot: BE02054725), rabbit polyclonal anti-AKT2 (lot: BE01031504), rabbit polyclonal anti-VEGFA (lot: BE02074125), and mouse monoclonal anti-CAMK2β (lot: BE02187240) (all from Bioss, Beijing, China; 1:1000 dilution); mouse monoclonal anti-GAPDH IgG (lot: 24040801, KMBR, Beijing, China; 1:1000 dilution) as loading control. After washing with TBST, membranes were incubated with horseradish peroxidase-conjugated secondary antibodies (1:1000 dilution) for 1 h at room temperature. Signals were visualized using the Western Lightning Plus-ECL system (PerkinElmer, Waltham, MA, USA) and quantified with ImageJ software (version 1.34, NIH).

### 4.13. Deep Learning-Based Drug–Target Interaction (DTIAM) Prediction

To systematically identify the bioactive components of YXYS targeting the HIF−1 signaling pathway and verify the multi-target mechanism, we employed DTIAM, a state-of-the-art, unified deep learning framework designed for high-precision DTI and binding affinity (DTA) prediction [43]. Unlike conventional docking methods that rely solely on 3D geometric fitting, DTIAM leverages self-supervised pre-training on massive unlabeled data to capture deep semantic features of molecular substructures and protein evolutionary patterns. The prediction workflow consisted of three advanced modules:

Molecular Representation Learning (BERMol): The chemical structures of YXYS-derived compounds (e.g., Ganosporelactone A) were converted into canonical SMILES. We utilized the BERMol (Bidirectional Encoder Representations of Molecular) module, which is pre-trained on ~1.6 million compounds from the ChEMBL database. Employing a Transformer-based architecture with multi-head self-attention, BERMol treats molecular substructures as “words” and the whole molecule as a “sentence.” Through self-supervised tasks—including Masked Language Modeling (MLM) and Molecular Functional Group Prediction (MFGP)—the model extracts rich contextual chemical features and functional group properties, overcoming the limitations of sparse labeled data in traditional methods.

Target Protein Representation (ESM-2): Protein sequences of the core targets (HIF−1α, RAGE, VEGF, and AKT2) were retrieved from the UniProt database. To capture the intrinsic evolutionary information and residue-level dependencies, we utilized ESM-2, a large-scale protein language model. ESM-2 extracts high-dimensional embeddings directly from primary sequences by modeling long-range residue interactions, ensuring robust feature extraction even for targets with complex conformational dynamics.

Unified Interaction Prediction via AutoML: To ensure the reliability and robustness of the predictions, the drug and target representations were integrated into a downstream prediction module based on Automated Machine Learning (AutoML). This module utilizes multi-layer model stacking and repeated k-fold bagging strategies to aggregate predictions from multiple base models (e.g., neural networks, gradient boosted trees). This ensemble approach minimizes overfitting and provides a stable, high-confidence interaction score.

The interaction potential was evaluated using three metrics: (1) Interaction Probability ($P_{DTI}$), reflecting the confidence of binary interaction; (2) Predicted Binding Affinity ($K_{d}/A_{raw}$), quantifying the binding strength; and (3) Integrated Prioritization Score ($S_{final}$) a weighted consensus metric optimized for ranking. Compounds were prioritized based on their ability to target HIF−1α, followed by multi-target profiling against the neuroimmune axis (RAGE/VEGF) to validate the “multi-component, multi-target” mechanism of YXYS.

### 4.14. Molecular Docking

Molecular docking was employed to predict the binding interactions between three principal compounds-identified via drug–target interaction (DTIAM) analysis-and their corresponding protein targets. The chemical structures of these compounds were retrieved from the PubChem database, while the crystal structures of the target proteins were obtained from the RCSB Protein Data Bank and the AlphaFold Protein Structure Database. Docking simulations were conducted using Molecular Operating Environment (MOE) software, version 2019.01. The binding sites were identified, and interaction profiles-including hydrogen bonds and other non-covalent interactions-were evaluated. Each compound-protein pair was docked five times to ensure consistency and reliability of the results. Binding affinities and interaction energies were used to rank potential binding conformations.

### 4.15. Molecular Dynamics Simulation

Molecular dynamics simulations using GROMACS 2020.03 with the AMBER99SB-ILDN force field assessed the stability of Ganosporelactone A binding to HIF−1α. Docked complexes were solvated in a TIP3P water box with counterions for charge neutrality. Energy minimization used the steepest descent algorithm, followed by NVT (300 K) and NPT (1 bar) equilibration. A 100 ns production run maintained NPT conditions with a 2 fs time step, LINCS bond constraints, and PME for electrostatics. Structural stability was analyzed via RMSD, RMSF, Rg, SASA, and hydrogen bond dynamics, while binding free energies were calculated using MM/GBSA.

### 4.16. Statistical Analysis

Behavioral scoring, histological image quantification, and Western blot densitometric analysis were performed by investigators blinded to group identity using coded samples, with group codes disclosed only after data extraction and statistical analysis were completed. Data analyses were conducted using IBM SPSS Statistics (version 21.0). Two-way ANOVA with LSD post hoc testing was used for normally distributed datasets, while the Kruskal–Wallis test served as a non-parametric alternative for non-normally distributed data. A *p*-value < 0.05 was considered significant. Graphs and data plots were generated with GraphPad Prism (version 8).

## 5. Conclusions

In conclusion, this study demonstrates that YXYS exerts its neuroprotective effects in AD by systematically restoring neuro-immune homeostasis. Our integrated multi-omics and computational approach identified the HIF−1 and AGE-RAGE signaling pathways as the central mechanistic hubs mediating this therapeutic action. YXYS ameliorates neuroinflammation by rebalancing central immune cell populations, thereby fostering a more protective neuroimmune environment and preserving neuronal integrity. The stable, high-affinity binding of its bioactive constituent, Ganosporelactone A, to HIF−1α provides a direct molecular basis for this critical cellular stress and hypoxia-response pathway effect. By bridging initial observations from human single-cell transcriptomics to validated multi-level regulation in vivo, our findings establish that YXYS’s efficacy stems from its capacity to coordinately modulate intertwined pathological processes. This work not only validates YXYS as a promising multi-target therapeutic strategy for AD but also establishes a powerful research paradigm for elucidating the complex pharmacology of traditional herbal medicines.

## Figures and Tables

**Table 1:** Identification of chemical constituents in YXYS.

No.	Ion Mode	RT (min)	Identification	Formula	Neutral Mass (Da)	Observed m/z	Mass Error (ppm)	Adducts	Measured	Compound CID	Chemical Class
1	ESI (+)	0.52	3, 4-dihydroxycinnamic acid	C_9_H_8_O_4_	180.0400	181.0623	3.7	[M−H]^+^	6342	689043	Phenolic acid
2	ESI (−)	0.55	Picroside I	C_24_H_28_O_11_	492.1631	537.1654	3.4	[M+HCOO]^−^	29,506	6440892	Glycoside
3	ESI (+)	0.61	Ginsenoside Rh4	C_36_H_60_O_8_	620.4288	621.4362	0.1	[M−H]^+^	177,799	21599928	Saponin
4	ESI (−)	0.76	Ginsenoside Re	C_48_H_82_O_18_	946.5501	945.5504	4.0	[M−H]^−^	110,829	441921	Saponin
5	ESI (−)	3.63	Sanggenon A	C_25_H_24_O_7_	436.1522	435.1422	−5.2	[M−H]^−^	145,557	156707	Flavonoid
6	ESI (−)	6.32	Morroniside	C_17_H_26_O_11_	406.1475	405.1397	0.4	[M−H]^−^	456,229	11284529	Glycoside
7	ESI (+)	6.78	Umbelliferone	C_9_H_6_O_3_	162.0317	163.0395	3.2	[M−H]^+^	5048	5281426	Coumarin
8	ESI (+)	7.04	Ganosporelactone A	C_30_H_40_O_7_	512.2774	513.2845	−0.3	[M−H]^+^	50,317	78384957	Terpenoid
9	ESI (−)	8.48	Ginsenoside Rg1	C_42_H_72_O_14_	800.4922	845.5073	−3.2	[M+HCOO]^−^	26,772	441923	Saponin
10	ESI (+)	8.53	Epi-Kansenone	C_30_H_48_O_2_	440.3654	441.3727	0.1	[M−H]^+^	318,636	21629616	Terpenoid
11	ESI (+)	9.85	Marsdenoside B	C_45_H_68_O_14_	832.4609	833.4532	−0.2	[M−H]^+^	59,602	101743838	Saponin
12	ESI (+)	9.93	Ganoderol A	C_30_H_46_O_2_	438.3498	475.3777	−1.0	[M−H]^+^	29,980	13934284	Terpenoid
13	ESI (+)	10.04	Quinquenoside R1	C_56_H_94_O_24_	1150.6135	1151.6135	−0.5	[M−H]^+^	155,412	101679657	Saponin
14	ESI (−)	10.21	Anemoside B	C_53_H_86_O_22_	1074.5611	1073.5561	−5.1	[M−H]^−^	103,762	11636713	Saponin
15	ESI (−)	10.23	Yemuoside YM14	C_52_H_82_O_21_	1042.5349	1087.5433	3.4	[M+HCOO]^−^	63,642	71338577	Saponin
16	ESI (+)	10.32	Yesanchinoside E	C_54_H_92_O_23_	1108.6029	1109.6089	−1.2	[M−H]^+^	164,905	11136943	Saponin
17	ESI (−)	10.36	Ginsenoside Rc	C_53_H_90_O_22_	1078.5924	1077.5883	3.0	[M−H]^−^	9278	12855889	Saponin
18	ESI (+)	10.45	Marsdenoside A	C_45_H_70_O_14_	834.4766	835.4834	−0.6	[M−H]^+^	58,336	11228220	Saponin
19	ESI (+)	11.64	Butylphthalide	C_12_H_14_O_2_	190.5994	193.1240	5.1	[M−H]^+^	591,019	61361	Phthalide
20	ESI (+)	12.91	Ligustilide	C_12_H_14_O_2_	190.5994	191.1102	1.4	[M−H]^+^	68,245	5319022	Phthalide
21	ESI (−)	15.24	Platycodigenin	C_30_H_48_O_7_	520.3400	519.3391	−0.7	[M−H]^−^	66,048	12314399	Terpenoid
22	ESI (+)	15.88	Panaxydol	C_17_H_24_O_2_	260.1776	261.1849	0.1	[M−H]^+^	7584	126312	Polyacetylene
23	ESI (−)	16.05	Ganoderic Acid H	C_32_H_44_O_9_	572.2985	571.2902	−1.8	[M−H]^−^	22,050	73657194	Terpenoid
24	ESI (+)	16.48	Quercetin	C_15_H_10_O_7_	302.0427	303.0531	2.5	[M−H]^+^	12,356	5280343	Flavonoid
25	ESI (+)	20.01	Ginsenoside Rs1	C_55_H_92_O_23_	1120.6029	1121.5999	−4.2	[M−H]^+^	6373	85044013	Saponin
26	ESI (−)	20.33	Sesquiterpenoid	C_15_H_24_O_2_	236.1776	279.2143	3.1	[M+HCOO]^−^	5518	139087999	Terpenoid
27	ESI (−)	25.47	Ganoderic Acid E	C_30_H_44_O_8_	532.3036	531.2958	−1.0	[M−H]^−^	6401	14109508	Terpenoid
28	ESI (−)	27.60	Artemitin	C_20_H_20_O_8_	384.1158	383.3413	−4.2	[M−H]^−^	21,930	5320351	Flavonoid
29	ESI (+)	29.75	Ganoderic Acid A	C_30_H_44_O_7_	516.3087	517.3160	−0.5	[M−H]^+^	13,458	471002	Terpenoid
30	ESI (−)	30.48	Tellimagrandin II	C41H30O26	938.1025	983.0928	−8.1	[M+HCOO]^−^	5884	151590	Tannin
31	ESI (+)	34.56	Notoginsenoside Fe	C_47_H_80_O_17_	916.5396	917.7272	3.3	[M−H]^+^	50,327	90657714	Saponin

**Table 2:** Special primers used in qRT-PCR.

	Forward Primer	Reverse Primer
IL-1β	TCGCAGCAGCACATCAACAAGAG	AGGTCCACGGGAAAGACACAGG
TNF-α	ATGTCTCAGCCTCTTCTCATTC	GCTTGTCACTCGAATTTTGAGA
IFN-γ	CTCCTCCTCCTCCTCCTC	CCTCATCCTCGTCTTCTACC
IL-10	CAGAGAAGCATGGCCCAGAA	GCTCCACTGCCTTGCTCTTA

## Data Availability

The core source code of the DTIAM framework is available at https://github.com/CSUBioGroup/DTIAM.git, accessed on 24 January 2026. The raw transcriptomic (RNA-seq) and proteomic (LC-MS/MS) datasets has been assigned the permanent DOI: https://doi.org/10.5281/zenodo.18829796.

## Supplementary Materials

The following supporting information can be downloaded at: https://www.mdpi.com/article/10.3390/ph19030502/s1. Figure S1. Single-cell transcriptomic profiling of AD hippocampal vasculature (A) Stacked bar plot showing the relative proportions of nine brain cell types per sample. (B) Bar plot showing the relative proportions of nine annotated brain cell types in AD versus control groups. (C) Top 10 terms from BP, CC, and MF categories are shown. (D) Differential expression analysis was performed across nine cell types. The plot highlights significantly upregulated and downregulated genes. HIF−1α is marked as a representative therapeutic target relevant to YXYS-mediated modulation. Figure S2. Network pharmacology analysis of YXYS in AD. (A) Venn diagram showing the overlap between AD-related targets and YXYS targets, with 496 shared targets identified. (B) Venn diagram illustrating the distribution of shared targets among six constituent herbs of YXYS: *Panax quinquefolium* L., *Ganoderma lucidum*, *Panax notoginseng*, *Cornus officinalis*, *Ligusticum chuanxiong*, and *Lycopus lucidus*. (C) GO enrichment analysis of shared targets categorized into BP, CC, and MF. Figure S3. Transcriptomic analysis results. (A) PCA plot showing the clustering of control, model, and YXYS-treated groups. (B) Volcano plot of differentially expressed genes (DEGs) between model and control groups, with red points indicating upregulated genes, blue points for downregulated genes, and gray points for non-significant genes. (C) Venn diagram displaying the overlap of DEGs among control vs. YXYS, model vs. YXYS, and control vs. model comparisons. (D) COG classification of DEGs. (E-G) GO enrichment analyses of the DEGs, detailing biological processes, cellular components, and molecular functions. Figure S4. Transcriptomic analysis of neurovascular signaling and immune cell infiltration (A) Heatmap illustrating the expression levels of genes associated with the AGE-RAGE signaling pathway across the experimental groups. (B) Heatmap depicting the differential expression of genes in the HIF−1 signaling pathway among the groups. (C) GSEA plot for the TGF-beta signaling pathway. (D) GSEA plot for the VEGF signaling pathway. (E) Correlation matrix representing the relationships between various immune cell types within the experimental groups, with red indicating positive correlations and blue indicating negative correlations. Figure S5. Proteomic analysis and Integrated multi-omics analysis. (A) 3D PCA plot illustrating the clustering of control, model, and YXYS-treated groups based on protein expression. (B) Bar chart showing the DEPs statistics, including upregulated and downregulated genes, between control vs model and treatment vs model groups. (C) Heatmap of log_2_FC values for DEPs in YXYS vs. model and control vs. model group. Red indicates upregulation, while blue indicates downregulation. (D) GO enrichment analysis of DEPs. Figure S6. GO enrichment analysis of the 142 proteins screened based on DTI scores.

## Abbreviations

The following abbreviations are used in this manuscript:

Aβ	Amyloid beta
AD	Alzheimer’s disease
AKT2	AKT serine/threonine kinase 2
CAMK2B	Calcium/calmodulin-dependent protein kinase type II subunit beta
HIF−1α	Hypoxia-inducible factor 1-alpha
MD	Molecular dynamics
MMP	Mitochondrial membrane potential
MWM	Morris water maze
ROS	Reactive oxygen species
snRNA-seq	Single-nucleus RNA sequencing
VEGF	Vascular endothelial growth factor
WB	Western blot
YXYS	Yixin Yangshen Granules
