# Supplementary figures and images for: Yixin Yangshen Granules Target HIF−1 Signaling to Modulate the Neuroimmune Microenvironment in Alzheimer’s Disease: Insights from Integrative Multi-Omics and Deep Learning
Source: Pharmaceuticals (Basel). 2026 Mar 18;19(3):502. doi: 10.3390/ph19030502 (PMC13028915; doi:10.3390/ph19030502)

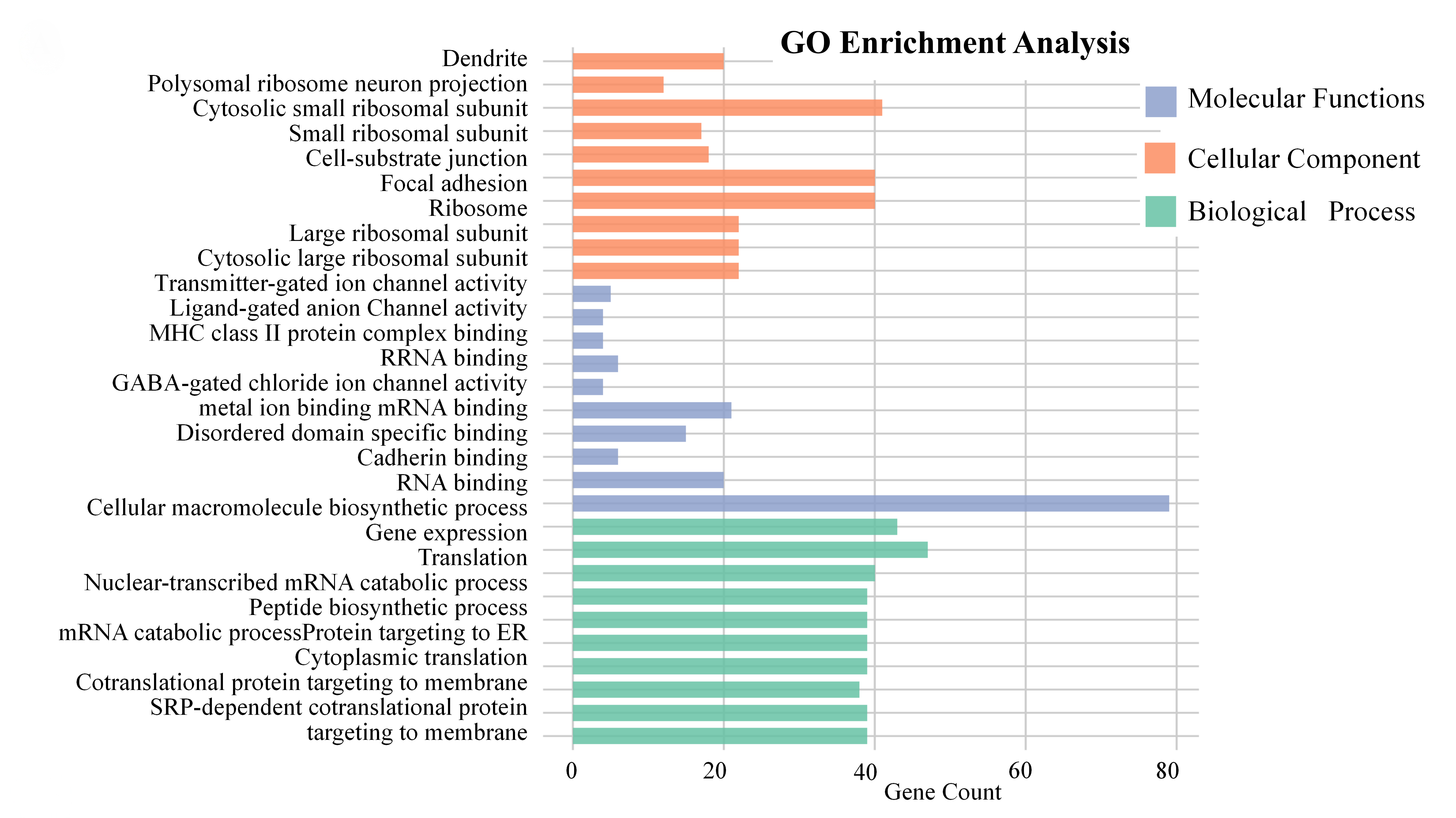

Supplement: Supplementary file 1 [file pharmaceuticals-19-00502-s001.zip › Figure S6.png]

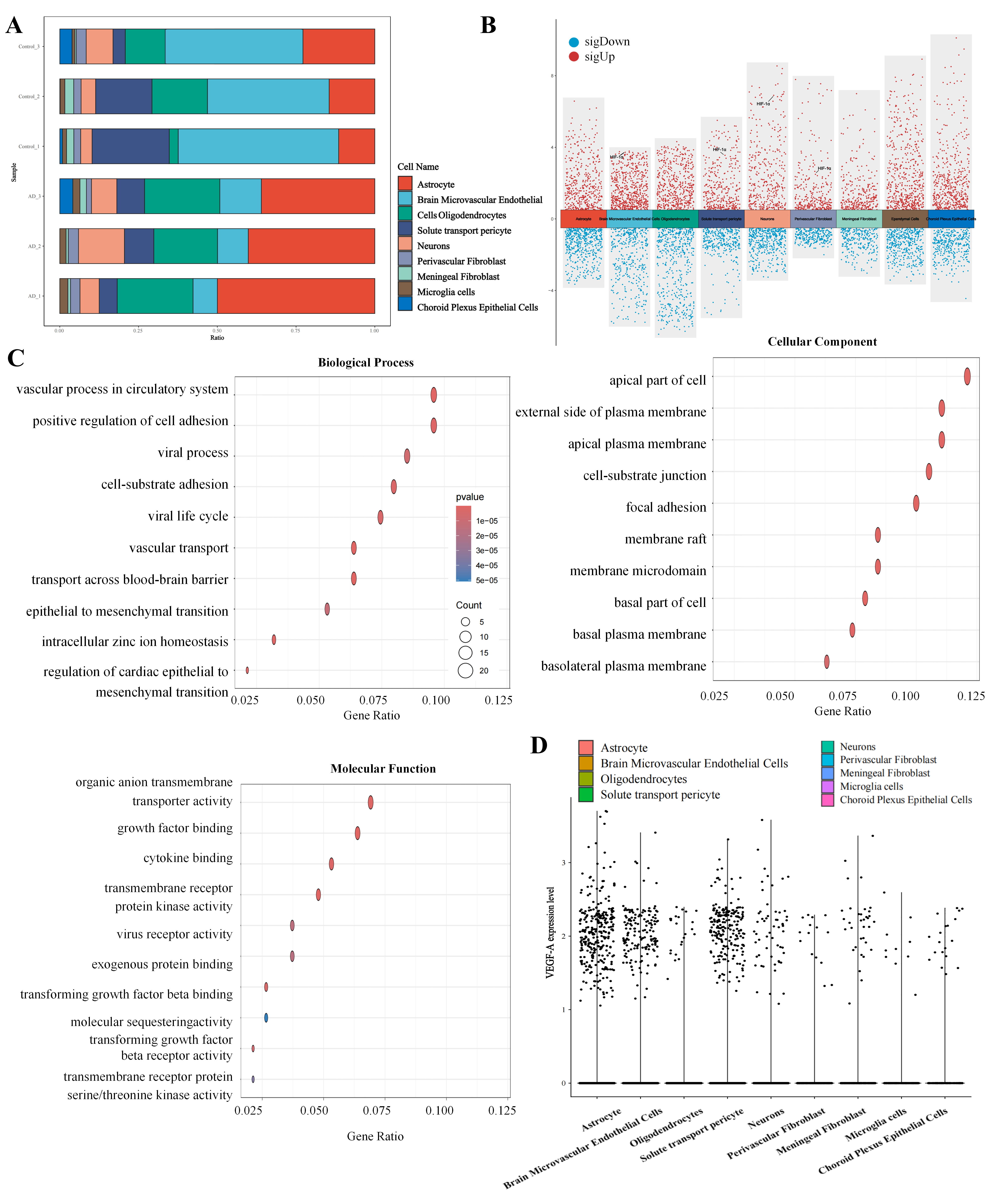

Supplement: Supplementary file 1 [file pharmaceuticals-19-00502-s001.zip › Figure S1.png]

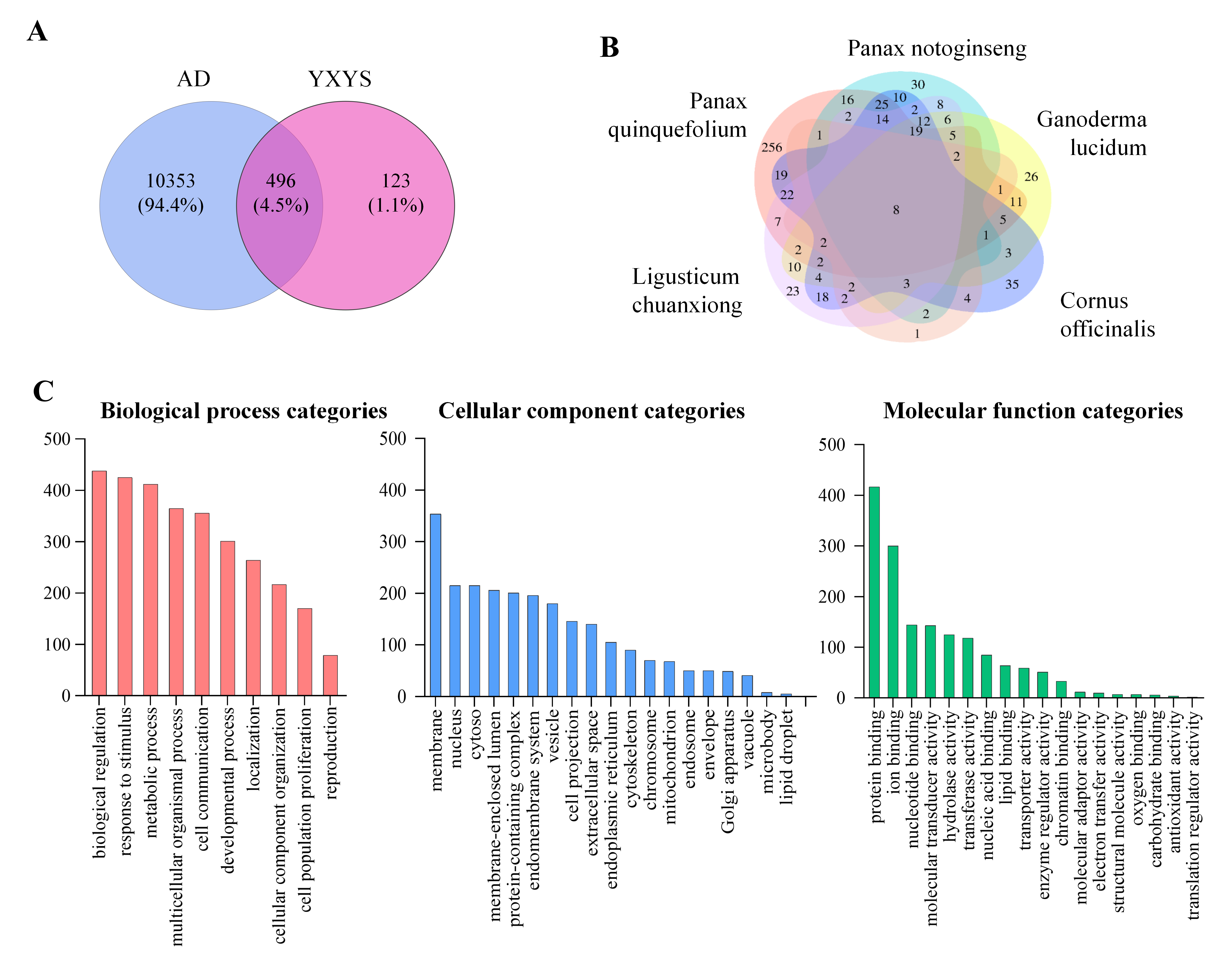

Supplement: Supplementary file 1 [file pharmaceuticals-19-00502-s001.zip › Figure S2.png]

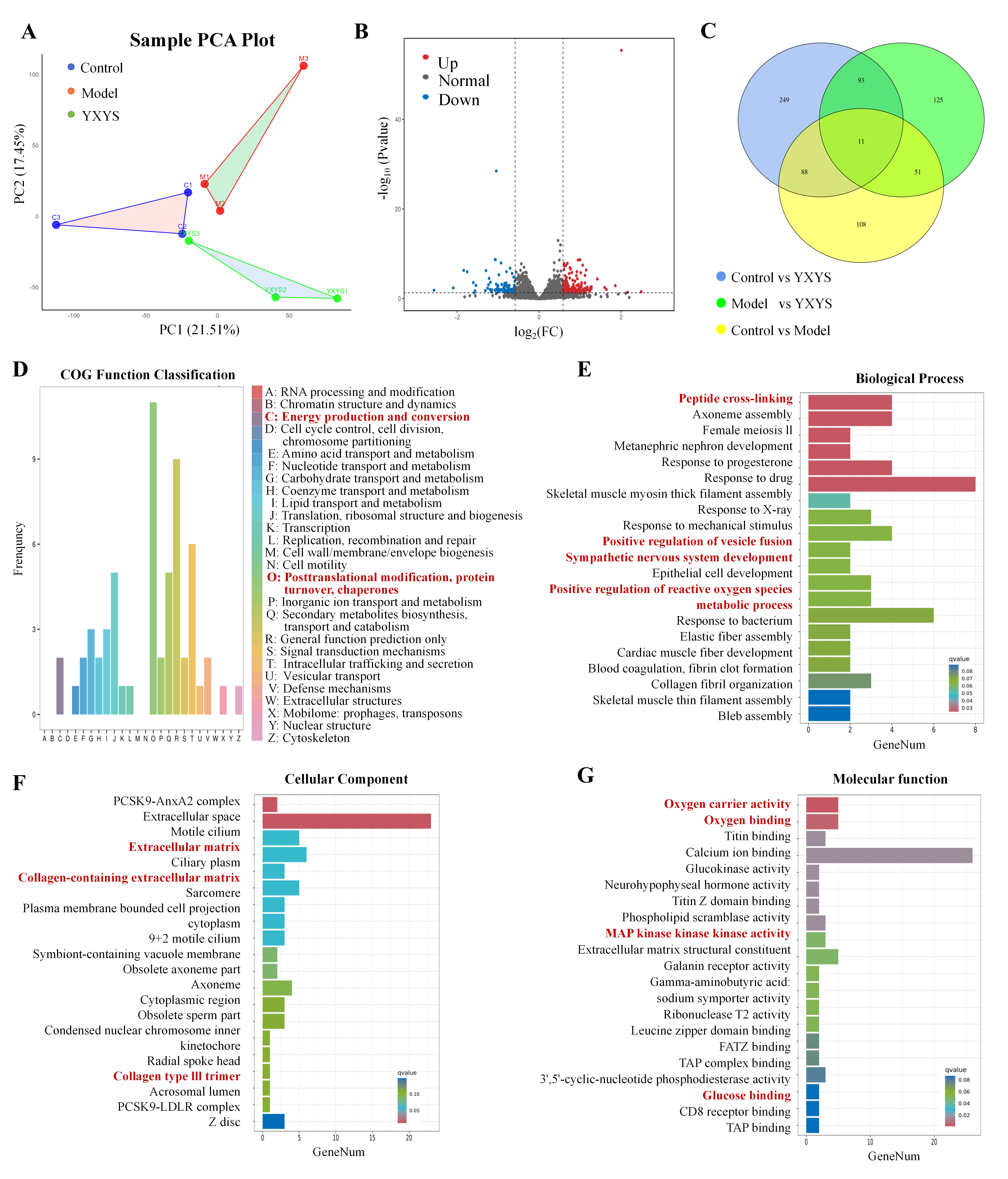

Supplement: Supplementary file 1 [file pharmaceuticals-19-00502-s001.zip › Figure S3.png]

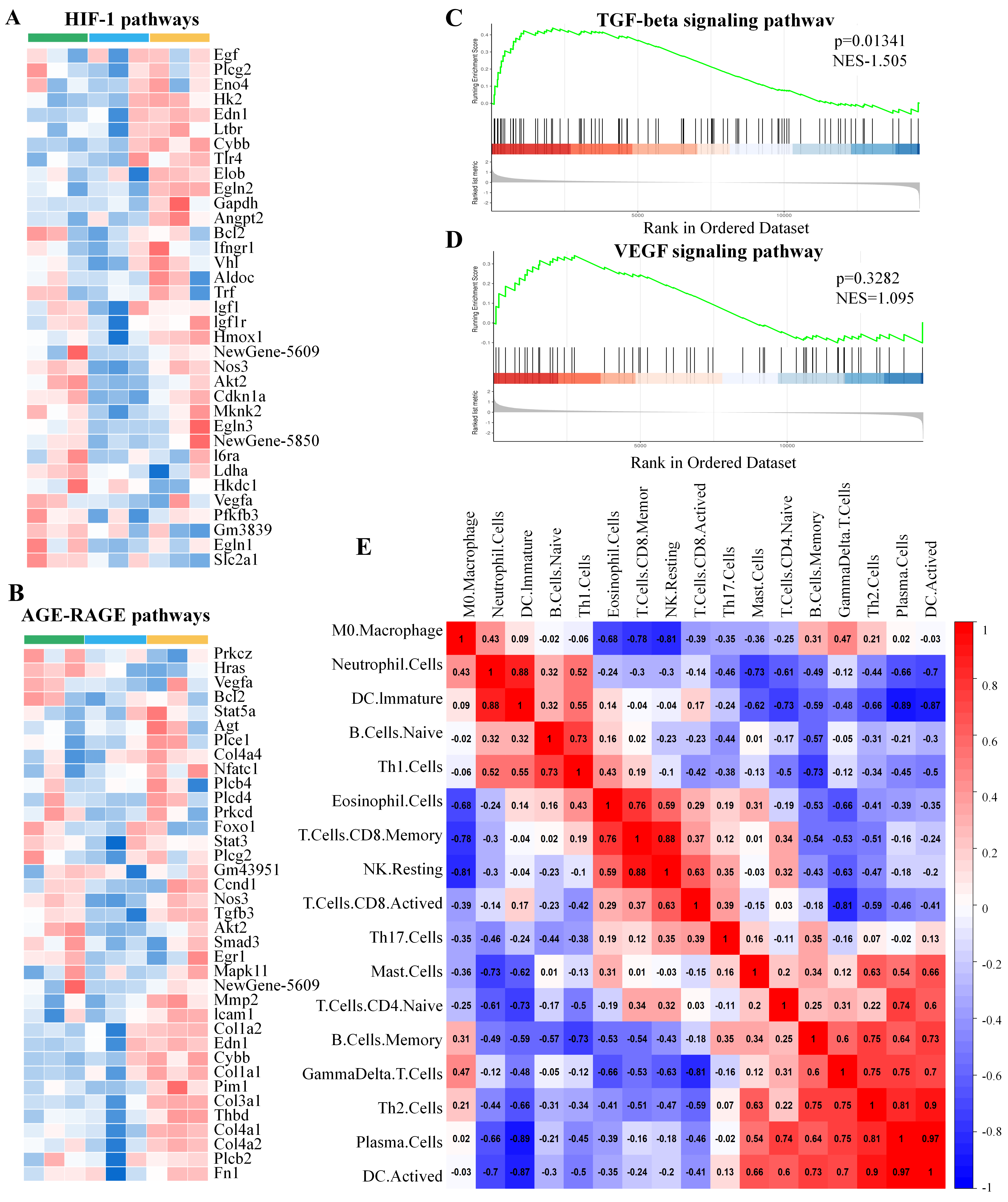

Supplement: Supplementary file 1 [file pharmaceuticals-19-00502-s001.zip › Figure S4.png]

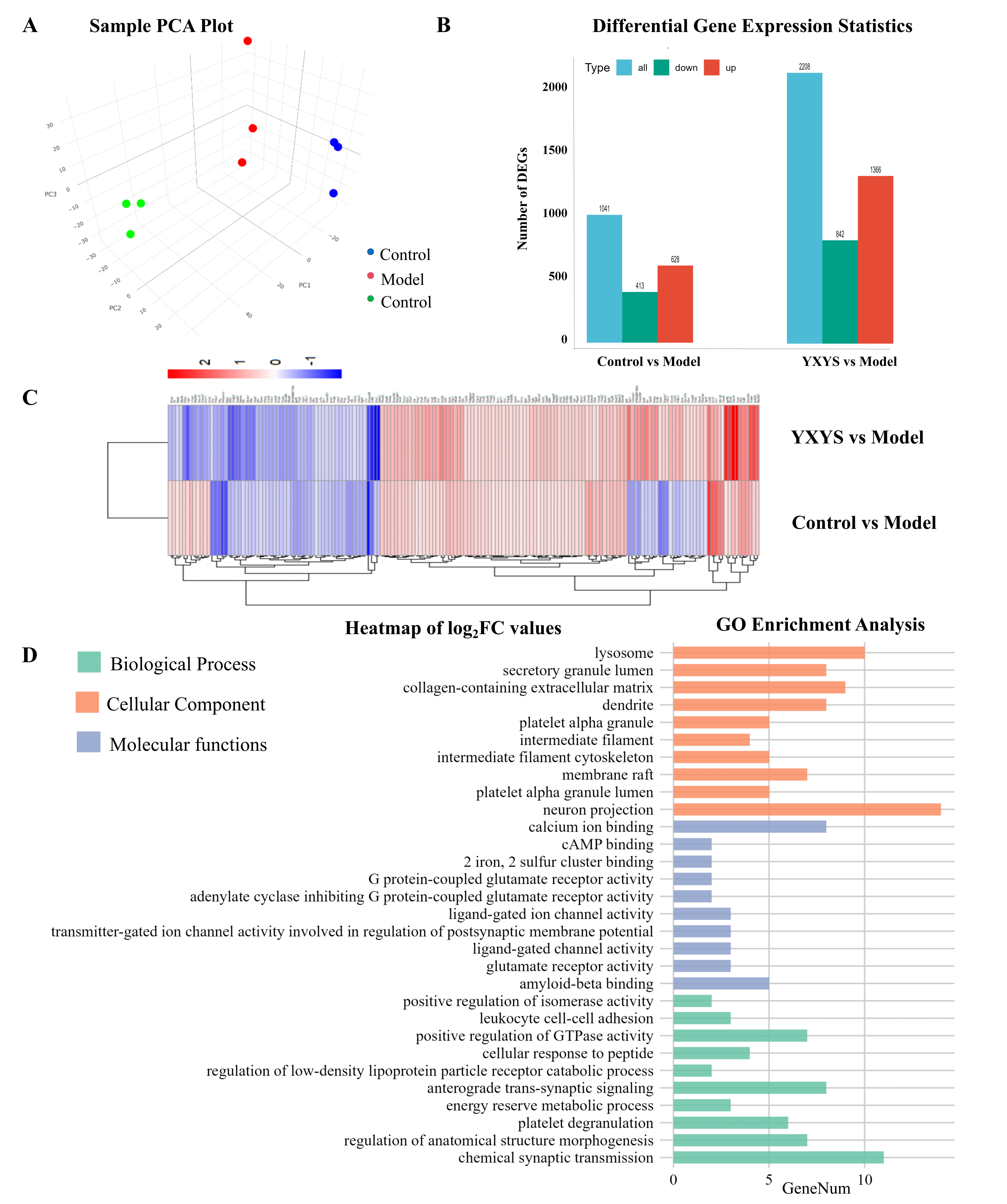

Supplement: Supplementary file 1 [file pharmaceuticals-19-00502-s001.zip › Figure S5.jpg]
